# Supplementary material for: Minimally invasive surgical techniques for oesophageal cancer and nutritional recovery: a prospective population-based cohort study
Source: BMJ Open. 2022 Sep 1;12(9):e058763. doi: 10.1136/bmjopen-2021-058763 (PMC9438117; doi:10.1136/bmjopen-2021-058763)
Supplement: Supplementary data [file bmjopen-2021-058763supp001.pdf]

Supplementary data to Minimally invasive surgical techniques for esophageal cancer and nutritional recovery – a prospective population-based cohort study.

Supplementary Table 1

Supplementary Table 2

Supplementary Figure 1

Supplementary Table 1. The proportion of minimally invasive esophagectomy, hybrid minimally invasive esophagectomy and open esophagectomy in the present study (OSCAR, Oesophageal Surgery on Cancer patients–Adaptation and Recovery), and in the population-based nationwide study of patients undergoing esophagectomy in Sweden (SESS, Swedish Esophageal Cancer Surgery Study) during the same time period.

	OSCAR-study	SESS-study
**Surgical approach**		
Minimally invasive	28 (21.7%)	76 (14.9%)
Hybrid minimally invasive	37 (28.6%)	133 (27.6%)
Open surgery	64 (49.6%)	273 (56.6%)

Supplementary Table 2. Explanatory analysis of anastomotic leak and intensive care unit and hospital stay on associations between surgical technique for esophagectomy due to cancer and nutritional status and weight loss 1 year after surgery in a Swedish population-based cohort study

	Open esophagectomy	Minimally invasive esophagectomy	Hybrid minimally invasive esophagectomy
	(Reference)	OR (95% CI)[1]	OR (95% CI)[1]
**Malnutrition – total score from abPG-SGA**	1.0	1.80 (0.84-3.86)	0.90 (0.39-2.07)
Reduced food intake	1.0	3.25 (1.60-6.57)	1.46 (0.73-2.93)
Symptoms reducing food intake[2]	1.0	1.35 (0.66-2.77)	0.96 (0.46-2.02)
Decreased activities/function	1.0	1.44 (0.48-4.35)	0.40 (0.09-1.77)
Weight			
Weight loss 6 months after surgery			
≤10%	1.0	1.14 (0.31-4.12)	1.04 (0.28-3.83)
>10%	1.0	1.49 (0.42-5.30)	1.56 (0.44-5.55)
Weight loss 1 year after surgery			
≤10%	1.0	2.86 (0.74-11.11)	0.73 (0.23-2.38)
>10%	1.0	1.56 (0.42-5.77)	0.84 (0.28-2.51)

[1] Adjusted for age, sex, Charlson's comorbidity index, preoperative BMI, pathological tumor stage, neoadjuvant therapy, enteral/parenteral nutrition support, postoperative complications, anastomotic leak, intensive care unit stay, and hospital stay.

[2] Nausea, diarrhoea, dry mouth, problems swallowing, feel full quickly, fatigue, pain etc.

Abbreviations: OR, Odds ratio; CI, confidence interval.

Supplementary Figure 1

Scored Patient-generated Subjective Global Assessment (PG-SGA)

History: Boxes 1 - 4 are designed to be completed by the patient.
[Boxes 1-4 are referred to as the PG-SGA Short Form (SF)]

Patient identification information

1. Weight *(see worksheet 1)*

In summary of my current and recent weight:

I currently weigh about _____ pounds
I am about _____ feet _____ inches tall

One month ago I weighed about _____ pounds
Six month ago I weighed about _____ pounds

During the past two weeks my weight has:

☐ Decreased (1) ☐ Not changed (0) ☐ Increased (0)

Box 1 ☐

2. Food intake: As compared to my normal intake, I would rate my food intake during the past month as

☐ Unchanged (0)
☐ More than usal (0)
☐ Less than usal (1)

I am now taking

☐ *Normal food* but less than normal amount (1)
☐ Little solid food (2)
☐ Only liquids (3)
☐ Only nutritional supplements (3)
☐ Very little of anything (4)
☐ Only tube feedings or only nutribution by vein (0)

Box 2 ☐

3. Symptoms: I have had the following problems that have kept me from eating enough during the past two weeks (check all that apply)

☐ No problem eating (0)
☐ No appetite, just did not feel like eating (3)
☐ Vomiting (3)
☐ Nausea (1)
☐ Diarrhea (3)
☐ Constipation (1)
☐ Dry moth (1)
☐ Mouth sores (2)
☐ Smells bother me (1)
☐ Things taste funny or have no taste (1)
☐ Feel full quickly (1)
☐ Problems swallowing (2)
☐ Fatigue (1)
☐ Pain; where?(3) ________________
☐ Other(1)** ________________

Examples: Depression,money, or dental problems **Box 3 ☐

4. Activities and function:
Over the past month, I would generally rate my activity as:

☐ Normal with no limitations (0)
☐ Not my normal self, but able to be up and about with fairly normal activities (1)
☐ Not feeling up to most things, but in bed or chair less than half the day (2)
☐ Able to be little activity and spend most of the day in bed or chair (3)
☐ Pretty much bed ridden, rarely out of bed (3)

Box 4 ☐

The remainder of this form is to be completed by your doctor, nurse, dietitian, or therapist. Thank you.

Additive score of boxes 1-4 ☐ **A**

©FD Ottery 2005, 2006, 2015 v3.22.15
email: faithotterymdphd@aol.com or info@pt-global.org
