# Supplementary material for: Minimally invasive surgical techniques for oesophageal cancer and nutritional recovery: a prospective population-based cohort study
Source: BMJ Open. 2022 Sep 1;12(9):e058763. doi: 10.1136/bmjopen-2021-058763 (PMC9438117; doi:10.1136/bmjopen-2021-058763)
Supplement: Author's manuscript [file bmjopen-2021-058763.draft_revisions.pdf]

BMJ Open is committed to open peer review. As part of this commitment we make the peer review history of every article we publish publicly available.

When an article is published we post the peer reviewers' comments and the authors' responses online. We also post the versions of the paper that were used during peer review. These are the versions that the peer review comments apply to.

The versions of the paper that follow are the versions that were submitted during the peer review process. They are not the versions of record or the final published versions. They should not be cited or distributed as the published version of this manuscript.

BMJ Open is an open access journal and the full, final, typeset and author-corrected version of record of the manuscript is available on our site with no access controls, subscription charges or pay-per-view fees (http://bmjopen.bmj.com).

If you have any questions on BMJ Open's open peer review process please email info.bmjopen@bmj.com

BMJ Open

Minimally invasive surgical techniques for esophageal cancer and nutritional recovery – a prospective population-based cohort study.

Journal:	*BMJ Open*
Manuscript ID	bmjopen-2021-058763
Article Type:	Original research
Date Submitted by the Author:	28-Oct-2021
Complete List of Authors:	Kauppila, Joonas; Karolinska University Hospital, Department of Molecular medicine and Surgery; University of Oulu, Department of Surgery<br>Rosenlund, Helen; Karolinska University Hospital, Department of Molecular medicine and Surgery; Danderyds Sjukhus AB, Department of Orthopaedics<br>Klevebro, Fredrik; Karolinska Institutet, Surgical Care Science<br>Johar, Asif; Karolinska Institutet, Department of Molecular medicine and Surgery<br>Anandavadivelan, Poorna; Karolinska Institutet, Department of Molecular medicine and Surgery<br>Mälberg, Kalle; Karolinska Institutet, Upper Gastrointestinal Research, Department of Molecular Medicine and Surgery<br>Lagergren, Pernilla; Karolinska Institute, Department of Molecular Medicine and Surgery
Keywords:	Oesophageal disease < GASTROENTEROLOGY, SURGERY, NUTRITION & DIETETICS

SCHOLARONE™
Manuscripts

I, the Submitting Author has the right to grant and does grant on behalf of all authors of the Work (as defined in the below author licence), an exclusive licence and/or a non-exclusive licence for contributions from authors who are: i) UK Crown employees; ii) where BMJ has agreed a CC-BY licence shall apply, and/or iii) in accordance with the terms applicable for US Federal Government officers or employees acting as part of their official duties; on a worldwide, perpetual, irrevocable, royalty-free basis to BMJ Publishing Group Ltd ("BMJ") its licensees and where the relevant Journal is co-owned by BMJ to the co-owners of the Journal, to publish the Work in this journal and any other BMJ products and to exploit all rights, as set out in our licence.

The Submitting Author accepts and understands that any supply made under these terms is made by BMJ to the Submitting Author unless you are acting as an employee on behalf of your employer or a postgraduate student of an affiliated institution which is paying any applicable article publishing charge ("APC") for Open Access articles. Where the Submitting Author wishes to make the Work available on an Open Access basis (and intends to pay the relevant APC), the terms of reuse of such Open Access shall be governed by a Creative Commons licence – details of these licences and which Creative Commons licence will apply to this Work are set out in our licence referred to above.

Other than as permitted in any relevant BMJ Author's Self Archiving Policies, I confirm this Work has not been accepted for publication elsewhere, is not being considered for publication elsewhere and does not duplicate material already published. I confirm all authors consent to publication of this Work and authorise the granting of this licence.

For peer review only

Minimally invasive surgical techniques for esophageal cancer and nutritional recovery – a prospective population-based cohort study.

Joonas H. Kauppila PhD[1,2], Helen Rosenlund PhD[1,3], Fredrik Klevebro PhD[1], Asif Johar MSc[1], Poorna Anandavadivelan PhD[1], Kalle Mälberg MSc[1], Pernilla Lagergren PhD[1,4]

[1] Surgical Care Sciences, Department of Molecular medicine and Surgery, Karolinska Institutet, Karolinska University Hospital 171 76 Stockholm, Sweden.

[2] Department of Surgery, Oulu University Hospital, University of Oulu, Finland

[3] Clinical Nutrition and Dietetics, Department of Orthopaedics, Danderyd Hospital, Stockholm, Sweden.

[4] Department of Surgery & Cancer, Imperial College London, United Kingdom

Corresponding author and requests for reprints:

Joonas H. Kauppila, Surgical Care Science, Department of Molecular medicine and Surgery, Karolinska Institutet, Karolinska University Hospital, Retzius väg 13A, 171 77 Stockholm, Sweden; E-mail: joonas.kauppila@ki.se

Running title: Surgical techniques and nutritional recovery.

Funding: The Swedish Cancer Society, the Stockholm County Council, and the Cancer Research Funds of Radiumhemmet.

Conflict of interest: All authors declare that they have no conflict of interest in relation to this manuscript.

For peer review only

ABSTRACT (Word count 283)

Objectives: To explore whether the minimally invasive (MIE), or hybrid minimally invasive esophagectomy (HMIE) are associated with better nutritional status and less weight loss 1 year after surgery, compared with open esophagectomy (OE).

Design: Prospective cohort study

Setting: All patients undergoing esophagectomy for cancer in Sweden during 2013-2018.

Participants: A total of 424 patients alive at 1 year after surgery were eligible, and 281 completed the 1-year assessment. Of these, 239 had complete clinical data and were included in the analysis.

Primary and secondary outcome measure: The primary outcome was nutritional status at 1 year after surgery, assessed using the abPG-SGA questionnaire. The secondary outcomes included postoperative weight loss at 6 months and 1 year after surgery.

Results: Of the included patients, 78 underwent MIE, 74 HMIE while 87 patients underwent OE. The MIE group had the highest prevalence of malnutrition (42% vs. 22% after HMIE vs. 25% after OE), reduced food intake (63% vs 45% after HMIE vs. 39% after OE), symptoms reducing food intake (60% vs. 45% after HMIE vs. 60% after OE) and abnormal activities/function (45% vs. 32% after HMIE vs 43% after OE). After adjustment for confounders, MIE was associated with a statistically significant increased risk of reduced food intake 1 year after surgery (OR 2.87, 95% CI 1.47-5.61), compared to OE. Other outcomes were not statistically significantly different between the groups. No statistically significant associations were observed between surgical techniques and weight loss up to 1 year after surgery.

Conclusions: MIE was statistically significantly associated with reduced food intake 1 year after surgery. However, no differences were observed in weight loss between the surgical

For peer review only

techniques. Further studies on nutritional impact of surgical techniques in esophageal cancer are needed.

Keywords: esophageal cancer; minimally invasive surgery; nutrition

Strengths and limitations of this study:

- The strength of this study is the prospective and population-based study design with longitudinal collection of data according to a predefined study protocol.

- The relatively high inclusion rate and complete data are strengths.

- The abPG-SGA questionnaire is validated and commonly used to measure malnutrition in oncology patients.

- A weakness of this study was that all patients who died during 1 year after esophagectomy were not eligible.

For peer review only

INTRODUCTION

The mainstay of curative treatment for esophageal cancer is surgery which carries a high risk of postoperative complications and mortality (1). The surgery is often combined with preoperative neoadjuvant therapy (2). The traditional open esophagetomy (OE) with abdominal and thoracic incisions is being replaced by minimally invasive esophagectomy (MIE), performed using thoracoscopy and laparoscopy (3). MIE is associated with lower risk of postoperative complications, especially pulmonary, lower in-hospital mortality, shorter length of hospital stay, and lower 90-day mortality, compared to OE (4-7). MIE has also been associated with improved health-related quality of life (HRQL) (8, 9). Hybrid minimally invasive esophagectomy (HMIE) applies open surgery in combination with laparoscopic or thoracoscopic technique, e.g. a thoracotomy and laparoscopy. HMIE is associated less postoperative mortality and better HRQL, compared with OE (10).

Esophageal cancer is associated with significant nutritional problems and weight loss before and after treatment (11-13). Patients lose between 5-12% of their preoperative weight during the first 6 months after esophagectomy and a majority have lost more than 10% of their body weight 12 months postoperatively (14). However, the effect of MIE on nutritional recovery remains unclear. It was hypothesized that MIE could improve nutritional recovery after esophagectomy, compared to OE.

The aim of this study was to investigate the impact of surgical technique, used for esophagectomy for cancer, on nutritional status and weight loss up to 1 year after surgery in a population-based and nationwide setting.

For peer review only

MATERIALS AND METHODS

Study design

This project was based on the nationwide and prospective cohort entitled Oesophageal Surgery on Cancer patients–Adaptation and Recovery (OSCAR) (15). Patients who underwent surgery for esophageal cancer in Sweden from 1st January 2013 and onwards were included and first inclusion started 1st January 2014. Eligible patients were identified from pathology units at eight treating hospitals in Sweden. An invitation letter was sent and within 1 week patients were contacted by telephone to get information regarding the project and to give their consent to participate. Arrangements for the first assessment were also made. A research nurse visited the patients in their homes for collection of patient-reported outcomes, objective measures of body weight and body composition. The OSCAR study was approved by the Regional Ethical Review Board in Stockholm, Sweden and all participants provided written informed consent.

view only

Clinical data at time of surgery was collected from medical records, which were reviewed according to a predefined study protocol to ensure uniformity: 1) Patient characteristics including age, sex, comorbidities, and weight at time of surgery; 2) Tumor histology, site/stage; 3) Treatment, including neoadjuvant or adjuvant oncological treatment, and surgical approach; 4) Postoperative complications classified according to the Clavien-Dindo scoring system (16).

Exposures

The following three types of surgery were used as exposures: 1) open transthoracic or transhiatal esophagectomy (OE; reference group), 2) minimally invasive esophagectomy (thoracoscopic-laparoscopic) (MIE), and 3) hybrid minimally invasive esophagectomy

(thoracoscopic/open abdomen or laparoscopic/open chest) (HMIE). Data on surgical technique was obtained from the medical records.

Outcomes

The primary outcome was malnutrition measured by the abPG-SGA (abbreviated patient-generated subjective global assessment) questionnaire (Appendix 1), that was completed by the patient at one time-point 1 year after surgery. This is a screening tool to detect patients at risk of malnutrition and is recommended for use in oncology patients (17, 18). The abPG-SGA is an abbreviated version of the PG-SGA questionnaire and has been found valid to detect malnutrition when used in an outpatient oncology clinic (19).

The abPG-SGA contains four sections which concern body weight, food intake, symptoms reducing food intake, and activities/function. The first section about body weight asks for the patient's current weight and height, weight at 1 and 6 months ago, and if the patient's weight has "decreased"/"increased"/"has been stable" during the last two weeks. The second section concerns food intake where the patient compares his/her food intake to their normal intake as "unchanged"/"more than usual"/"less than usual". The patient is then asked to relate his/her food intake to the habitual food intake prior to cancer symptoms/diagnosis (in the original questionnaire patients related food intake to 1 month ago). If the answer was "less than usual" the patient responded to the follow-up question: What type of food do you eat? with the following response alternatives "normal food but less than normal amount"/"little solid food"/"only liquids"/"only nutritional supplements"/"very little of anything"/"only tube feeding or parenteral nutrition". Section three covers symptoms that have kept the patient from eating enough during the past two weeks (several answers possible): "no problem"/"no appetite, just did not feel like eating"/"nausea"/"vomiting"/"constipation"/

For peer review only

"diarrhoea"/"mouth sores"/"dry mouth"/"things taste funny or have no taste"/"smells bother me"/"problems swallowing"/"feel full quickly"/"pain"/"other". The forth section concerns activities/function and asks the patients to rate their general activity over the past months: "normal"/"not my normal self, but have fairly normal activities"/"not feeling up to things, in bed/chair half the day"/"able to be little active and spend most of the day in bed/chair"/"rarely out of bed". The score from each part totals up a sum that determines the patient´s nutritional status. A higher score indicates more severe malnutrition (17, 19).

The secondary outcomes were postoperative weight loss between 1) time of surgery and 6 months postoperatively and 2) time of surgery and 1 year after surgery. The weight at surgery was collected from medical records, weight at 6 months was reported by the patient in the abPG-SGA questionnaire, and weight at 1 year was measured by the research nurse. Weight loss was calculated by comparing weight measures at surgery and weight at 6 months and at 1 year.

Statistical analysis

Logistic regression models were used to analyze the associations between surgical techniques (OE [reference group], MIE, HMIE) and malnutrition (yes, no) and weight loss at 6 months and at 1 year postoperatively. Weight loss was categorized as 1) No weight loss/weight gain, 2) ≤10% weight loss, 3) >10% weight loss. Results were presented as odds ratios (OR) with 95% confidence intervals (CI), adjusted for potential confounders: age (longitudinal), sex (male, female), Charlson's comorbidity index (0, 1, ≥2), preoperative body mass index (BMI) (<25, ≥25), pathological tumor stage (0-I, II, III-IV), neoadjuvant therapy (yes, no), enteral/parenteral nutrition support during at least 1 week postoperatively (yes, no), and postoperative complications (Clavien-Dindo score [CDS]; low grade [0-II], high grade [III-

For peer review only

IV]). Weight loss was also presented as median weight loss percentage, combined with the inter quartile range for each group. In the analyses of the abPG-SGA questionnaire, a summarized score of ≥6 was considered as malnutrition. For body weight, food intake, symptoms reducing food intake, and activities/function a score of ≥2 was considered as a lower/worse condition in each group, with a reference score of <2. A P-value <0.05 was considered statistically significant.

Patient and public involvement

The Surgical Care Science research partnership group including patients with esophageal cancer were involved in the planning of OSCAR cohort study. The partnership group patients were involved in discussions around the study questions, as well as on the potential outcome measures collected during the study. The partnership group were not involved in recruitment of participants, but have been involved in the dissemination of research findings and increasing public awareness of esophageal cancer. Regarding the present study, the research partnership group patients suggested nutritional as an important outcome requiring more studies. Findings from this study will be communicated to patients and healthcare professionals through conferences and patient meetings.

For peer review only

RESULTS

Participants

Between January 2013 and April 2018, 675 patients underwent esophageal cancer surgery in Sweden. Of these, 511 (76%) survived for at least 1 year, 85 were not reachable, and 2 were excluded because of cognitive impairment, leaving 424 patients eligible for inclusion. Of these, 281 (66%) patients completed the 1-year assessment and clinical data were available for 247 (58%) patients. In total, 239 patients were included in the analyses.

Patient characteristics

Patients were evenly distributed in the 3 surgical groups (OE 36%, MIE 33%, and HMIE 31%) (Table 1). The mean age was 66.3 ($\pm$8.7) years at time of surgery and most patients were male (87%). The distribution of preoperative BMI and weight loss were similar across the three groups. Preoperative neoadjuvant therapy was most often used in the HMIE group (84%), while a higher pathological tumor stage was more frequently seen in the MIE group (37%), compared to the other groups. Almost all patients had a gastric conduit, while few patients in the OE group had an esophagojejunostomy. A neck anastomosis was more common in the MIE group, compared to the OE and HMIE groups. Patients who underwent HMIE less often received enteral/parenteral nutrition for at least 1 week, compared to the other groups. Patients with jejunostomy received enteral feeding for 14 days in median.

Nutritional status

One third (30%) of all patients had malnutrition 1 year postoperatively, based on the abPG-SGA (Table 2), and it was highest in the MIE group (42%), compared to the OE (25%) and the HMIE (22%) groups. The MIE group had the highest prevalence of reduced food intake

For peer review only

(63% vs 45% after HMIE vs. 39% after OE), symptoms reducing food intake (60% vs. 45% after HMIE vs. 60% after OE). The number of reported symptoms reducing food intake (each symptom counted once for each patient, one patient could have multiple symptoms) in the MIE group was higher in the MIE group (121), compared to HMIE (71) and OE (107). Furthermore, abnormal activities/function were more common after MIE (45% vs. 32% after HMIE vs 43% after OE). In multivariable analysis, the point estimate for the risk of malnutrition was high after MIE, compared to OE (OR_{adj} 1.86, 95% CI 0.91-3.82), but this difference was statistically non-significant. A statistically significant association was observed between MIE and reduced food intake at 1 year after surgery (OR_{adj} 2.87, 95% CI 1.47-5.61), compared to OE (Table 3). No associations were observed between surgical technique and symptoms reducing food intake or activities/function.

In a sensitivity analysis the level of anastomosis was included in the model, but the results remained the same, except that the point estimate of reduced food intake after MIE was increased to 3.13 (95% CI 1.53-6.39). Furthermore, in an attempt to minimize the risk that patients in the OE group reconstructed with jejunal conduit affected the outcomes, these 8 patients were excluded from the analyses, but the results remain unchanged.

Weight loss

The patients lost most weight during the first 6 months postoperatively in all groups and it was most pronounced after HMIE, compared to both OE and MIE. A similar pattern was observed for weight loss at 1 year after surgery (Figure 1). Surgical technique was not associated with weight loss at 6 months or 1 year after surgery (Table 3).

For peer review only

DISCUSSION

The results of this population-based nationwide cohort study suggest that patients operated with MIE and HMIE had no increased risk in malnutrition compared to OE, but those operated with MIE were more likely to have reduced food intake 1 year postoperatively, compared to OE. No association between surgical technique and weight loss up to 1 year postoperatively was present.

The strength of this study is the prospective and population-based study design with longitudinal collection of data. Clinical data at time of surgery was collected from medical records and reviewed according to a predefined study protocol to ensure consistency and uniformity of the data. The cohort had relatively high inclusion rate, and completeness of the abPG-SGA questionnaire in survivors 1 year postoperatively was very high. The abPG-SGA questionnaire is a validated and commonly-used instrument that has been reported to be a valid tool to measure malnutrition in oncology patients (19). The results may be generalizable to hospitals in Western countries where these surgical techniques are used. Missing data is in general low when collected by a research nurse in patients' homes. A weakness of this study was that all patients who died during 1 year after esophagectomy were not eligible. Moreover, the patients´ self-reported weight measure could be biased, although this is assumed to be a minor problem since keeping track of weight is a central concern among these patients. In the section about food intake in the abPG-SGA, the nurse asked these patients to relate their actual food intake to their habitual eating prior to diagnosis, instead of 1 month ago, to make it more suitable for this patient group. This may give a more correct picture of the nutritional status in this group since comparing to 1 month ago would not have shown any difference because their eating problems started at time of surgery or earlier.

For peer review only

Previous studies comparing malnutrition between MIE and OE are sparse, as very few have looked at malnutrition in the long term, and none using the abPG-SGA questionnaire. The previous studies assessing malnutrition have used weight loss or BMI as a proxy for nutritional status(20, 21) or laboratory values (6, 22). In an Italian case-control study, serum albumin levels were higher at day 1, 3, and 8 in patients undergoing HMIE, compared to OE.(22) An American study suggested earlier oral intake in patients who underwent MIE, compared to OE(6). The results from the current study suggest that MIE has a greater negative effect on the patients´ food intake, compared to OE, which is the opposite of what we hypothesized *a priori*. However, there was no significant difference in malnutrition, or other nutritional symptoms between the surgical techniques. Based on previous research, MIE should lead to less postoperative complications and shorter length of hospital stay, compared to OE (5-7). In the present study, major complications were similar between the groups and likely not a relevant factor in food intake symptoms. On the other hand, neck anastomosis is known to be associated with more anastomotic strictures, compared to chest anastomosis, which might affect the food intake(23). As the MIE group had more neck anastomoses than HMIE and OE groups, a sensitivity analysis adjusted for anastomotic location was conducted but the results were no different from the main analysis. The conduit used for reconstruction might also affect food intake, but excluding jejunal interposition grafts did not change the results. Anastomotic techniques (i.e., handsewn vs circular stapled vs. linear stapled) might also differ between MIE, HMIE and OE. For example, end-to-side stapled and hand-sewn, as well as linear stapled side-to-side anastomoses have been described in Sweden(24, 25). However, these differences remain speculative, as this data was not available, and further studies are needed. Lastly, early oral feeding is central to any Enhanced Recovery After Surgery (ERAS) programs, (26) and could impact the long-term nutritional recovery of these

For peer review only

patients. However, participation in ERAS programs was not available in the dataset. As esophageal cancer strongly affects the patients´ food intake before and after surgery, the associated weight loss is often substantial(27). Some previous studies have investigated how surgical technique affects patients´ weight in the long-term. A Swedish clinical study observed no difference in weight loss during the first postoperative year in patients who underwent MIE, compared to OE (20). Our results suggested a higher weight loss in the HMIE group, both at 6 months and 1 year postoperatively, but this difference was non-significant in the adjusted analyses. In addition to no nutritional differences between the surgical methods, the results could be explained by too small number of patients in the analysis. However, the weight loss in the OE group was increasing up to 1 year after surgery, and studies with longer-term follow-up might be warranted.

Even though there were no major differences in malnutrition between minimally invasive and open esophagectomy, a striking finding of this study is that one third (30%) of all included patients were malnourished at 1 year after surgery. This highlights the importance of nutritional support and long-term follow-up from the health care for patients who undergo esophagectomy. Since esophagectomy results in a permanent anatomical change of the upper gastrointestinal tract, besides nutritional strategies in the immediate postoperative phase such as early enteral feeding, nutritional interventions for postoperative complications, attention to long-term nutritional intake and status are also of high importance(27). While surgical technique might not be of high importance, the impact of anastomotic technique on nutrition in esophageal cancer patients should be further evaluated. With improved knowledge, the utility of long-term dietitian support in different patient groups could be clarified.

For peer review only

In conclusion, this population-based prospective cohort study suggests that while there was no increased risk of malnutrition between minimally invasive compared to open esophagectomy, MIE is associated to reduced food intake 1 year postoperatively. No significant differences were present between surgical technique and weight loss. Further studies on nutritional impact of surgical techniques in esophageal cancer are needed.

ACKNOWLEDGEMENTS

We thank all study participants and the members of the Surgical Care Science patient research partnership group for their contribution to the OSCAR study.

Contributorship statement

JHK, HR, FK, PA, and PL contributed with input in designing the study. Data collection and assembly of data were performed by KM and PL. Data analysis was conducted by AJ. All authors (JHK, HR, FK, AJ, PA, KM, and PL) contributed in interpreting the results, manuscript writing and approval of the final version of the manuscript.

Competing interests

The authors state no conflicts of interest.

Funding statement

We also would like to thank the Swedish Cancer Society (grant number 180685), the Cancer Research Funds of Radiumhemmet (grant number 171103), and the Stockholm County Council (grant number LS 2018–1157) for financial support. Pernilla Lagergren is supported by the NIHR Imperial Biomedical Research Centre (BRC) for her position at Imperial

For peer review only

College London, London, United Kingdom. The funding sources had no role in the design and conduct of the data collection, management, analysis and interpretation of the data or preparation review or approval of the manuscript.

Data Sharing Statement

All data underlying the findings are presented in the manuscript. The datasets generated and/or analyzed in the current study will not be publicly available due to the ethical review act but will be available from the principal investigators of the study on reasonable request.

For peer review only

REFERENCES

1. Markar SR, Karthikesalingam A, Low DE. Outcomes assessment of the surgical management of esophageal cancer in younger and older patients. Ann Thorac Surg. 2012;94(5):1652-8.
2. Lagergren J, Smyth E, Cunningham D, Lagergren P. Oesophageal cancer. Lancet. 2017;390(10110):2383-96.
3. Lazzarino AI, Nagpal K, Bottle A, Faiz O, Moorthy K, Aylin P. Open versus minimally invasive esophagectomy: trends of utilization and associated outcomes in England. Ann Surg. 2010;252(2):292-8.
4. Briez N, Piessen G, Torres F, Lebuffe G, Triboulet JP, Mariette C. Effects of hybrid minimally invasive oesophagectomy on major postoperative pulmonary complications. Br J Surg. 2012;99(11):1547-53.
5. Yibulayin W, Abulizi S, Lv H, Sun W. Minimally invasive oesophagectomy versus open esophagectomy for resectable esophageal cancer: a meta-analysis. World J Surg Oncol. 2016;14(1):304.
6. Tapias LF, Mathisen DJ, Wright CD, Wain JC, Gaissert HA, Muniappan A, et al. Outcomes With Open and Minimally Invasive Ivor Lewis Esophagectomy After Neoadjuvant Therapy. Ann Thorac Surg. 2016;101(3):1097-103.
7. Kauppila JH, Helminen O, Kyto V, Gunn J, Lagergren J, Sihvo E. Short-Term Outcomes Following Minimally Invasive and Open Esophagectomy: A Population-Based Study from Finland and Sweden. Ann Surg Oncol. 2018;25(1):326-32.
8. Kauppila JH, Xie S, Johar A, Markar SR, Lagergren P. Meta-analysis of health-related quality of life after minimally invasive versus open oesophagectomy for oesophageal cancer. Br J Surg. 2017;104(9):1131-40.
9. Maas KW, Cuesta MA, van Berge Henegouwen MI, Roig J, Bonavina L, Rosman C, et al. Quality of Life and Late Complications After Minimally Invasive Compared to Open Esophagectomy: Results of a Randomized Trial. World J Surg. 2015;39(8):1986-93.
10. Mariette C, Markar SR, Dabakuyo-Yonli TS, Meunier B, Pezet D, Collet D, et al. Hybrid Minimally Invasive Esophagectomy for Esophageal Cancer. N Engl J Med. 2019;380(2):152-62.
11. Haverkort EB, Binnekade JM, Busch OR, van Berge Henegouwen MI, de Haan RJ, Gouma DJ. Presence and persistence of nutrition-related symptoms during the first year following esophagectomy with gastric tube reconstruction in clinically disease-free patients. World J Surg. 2010;34(12):2844-52.
12. Anandavadivelan P, Lagergren P. Cachexia in patients with oesophageal cancer. Nat Rev Clin Oncol. 2016;13(3):185-98.
13. Bozzetti F. Nutritional support in patients with oesophageal cancer. Support Care Cancer. 2010;18 Suppl 2:S41-50.
14. Baker M, Halliday V, Williams RN, Bowrey DJ. A systematic review of the nutritional consequences of esophagectomy. Clin Nutr. 2016;35(5):987-94.
15. Schandl A, Johar A, Anandavadivelan P, Vikstrom K, Malberg K, Lagergren P. Patient-reported outcomes 1 year after oesophageal cancer surgery. Acta Oncol. 2020:1-7.
16. Clavien PA, Barkun J, de Oliveira ML, Vauthey JN, Dindo D, Schulick RD, et al. The Clavien-Dindo classification of surgical complications: five-year experience. Ann Surg. 2009;250(2):187-96.

For peer review only

17. Persson C, Sjoden PO, Glimelius B. The Swedish version of the patient-generated subjective global assessment of nutritional status: gastrointestinal vs urological cancers. Clin Nutr. 1999;18(2):71-7.
18. Bauer J, Capra S, Ferguson M. Use of the scored Patient-Generated Subjective Global Assessment (PG-SGA) as a nutrition assessment tool in patients with cancer. Eur J Clin Nutr. 2002;56(8):779-85.
19. Gabrielson DK, Scaffidi D, Leung E, Stoyanoff L, Robinson J, Nisenbaum R, et al. Use of an abridged scored Patient-Generated Subjective Global Assessment (abPG-SGA) as a nutritional screening tool for cancer patients in an outpatient setting. Nutr Cancer. 2013;65(2):234-9.
20. Ericson J, Lundell L, Klevebro F, Kamiya S, Nilsson M, Rouvelas I. Long-term weight development after esophagectomy for cancer-comparison between open Ivor-Lewis and minimally invasive surgical approaches. Dis Esophagus. 2018.
21. Hynes O, Anandavadivelan P, Gossage J, Johar AM, Lagergren J, Lagergren P. The impact of pre- and post-operative weight loss and body mass index on prognosis in patients with oesophageal cancer. Eur J Surg Oncol. 2017;43(8):1559-65.
22. Scarpa M, Cavallin F, Saadeh LM, Pinto E, Alfieri R, Cagol M, et al. Hybrid minimally invasive esophagectomy for cancer: impact on postoperative inflammatory and nutritional status. Dis Esophagus. 2016;29(8):1064-70.
23. van Workum F, van der Maas J, van den Wildenberg FJ, Polat F, Kouwenhoven EA, van Det MJ, et al. Improved Functional Results After Minimally Invasive Esophagectomy: Intrathoracic Versus Cervical Anastomosis. Ann Thorac Surg. 2017;103(1):267-73.
24. Rutegard M, Lagergren J, Rouvelas I, Lindblad M, Blazeby JM, Lagergren P. Population-based study of surgical factors in relation to health-related quality of life after oesophageal cancer resection. Br J Surg. 2008;95(5):592-601.
25. Nilsson M, Kamiya S, Lindblad M, Rouvelas I. Implementation of minimally invasive esophagectomy in a tertiary referral center for esophageal cancer. J Thorac Dis. 2017;9(Suppl 8):S817-S25.
26. Weimann A, Braga M, Carli F, Higashiguchi T, Hubner M, Klek S, et al. ESPEN guideline: Clinical nutrition in surgery. Clin Nutr. 2017;36(3):623-50.
27. Steenhagen E, van Vulpen JK, van Hillegersberg R, May AM, Siersema PD. Nutrition in peri-operative esophageal cancer management. Expert Rev Gastroenterol Hepatol. 2017;11(7):663-72.

For peer review only

FIGURE LEGENDS

Figure 1. Median (%) weight loss at 6 months and at 1 year after surgery in patients who underwent esophagectomy with different surgical techniques; OE (open esophagectomy), MIE (minimally invasive esophagectomy), HMIE (hybrid minimally invasive esophagectomy).

For peer review only

Table 1. Distribution of patient characteristics in relation to the surgical technique used for esophagectomy for cancer in a Swedish population-based cohort study.

	All	Open esophagectomy (OE)	Minimally invasive esophagectomy (MIE)	Hybrid minimally invasive esophagectomy (HMIE)
	Number (%)			
Number of patients	239 (100)	87 (36)	78 (33)	74 (31)
Sex (male)	207 (87)	69 (79)	70 (90)	68 (92)
**At time of surgery**				
Age (mean + 1 SD)	66.3 + 8.7	65.8 + 8.4	66.2 + 9.8	66.9 + 7.9
Preoperative BMI (mean + 1 SD)				
<25	36 (15)	15 (17)	12 (15)	9 (12)
≥25	203 (63)	72 (83)	66 (85)	65 (88)
Preoperative weight loss				
No weight loss/weight gain	23 (10)	10 (11)	4 (5)	9 (12)
<10%	79 (33)	26 (30)	32 (41)	21 (28)
≥10%	137 (57)	51 (59)	42 (54)	44 (59)
Preoperative neoadjuvant treatment	188 (79)	68 (78)	58 (74)	62 (84)
Pathological tumor stage				
0-I	79 (33)	32 (37)	16 (21)	31 (42)
II	80 (33)	26 (30)	33 (42)	21 (28)
III-IV	80 (33)	29 (33)	29 (37)	22 (30)
Organ substitute				
Gastric tube	219 (92)	73 (84)	74 (95)	72 (97)
Esophagojejunostomy	10 (4)	8 (9)	1 (1)	1 (1)
Colonic interposition	2 (1)	1 (1)	1 (1)	0 (0)
Level of anastomosis				
Neck	38 (16)	9 (10)	20 (26)	9 (12)
Thorax	187 (78)	70 (80)	54 (69)	63 (85)
Abdomen	6 (3)	5 (6)	0 (0)	1 (1)
Charlson's comorbidity index				
0	116 (54)	43 (49)	37 (47)	36 (49)
1	77 (28)	27 (31)	23 (29)	27 (36)
≥2	46 (18)	17 (20)	18 (23)	11 (15)
Enteral/parenteral nutrition	175 (73)	68 (78)	60 (77)	47 (64)
Postoperative complications				
Low grade (CDS 0-II)	144 (60)	51 (59)	44 (56)	49 (66)
High grade (CDS III-IV)	95 (40)	36 (41)	34 (44)	25 (34)

SD=Standard Deviation, BMI=Body Mass Index, CDS=Clavien-Dindo Score.

For peer review only

Table 2. Results of the abPG-SGA questionnaire completed 1 year after esophagectomy for cancer in relation to surgical technique in a Swedish population-based cohort.

AbPG-SGA	All	Open esophagectomy (OE)	Minimally invasive esophagectomy (MIE)	Hybrid minimally invasive esophagectomy (HMIE)
	Number (%)			
Number of patients	239	87	78	74
Body weight				
Reduced	25 (10)	11 (13)	7 (9)	7 (9)
Not changed	173 (72)	64 (74)	51 (65)	58 (78)
Increased	39 (16)	10 (11)	20 (26)	9 (12)
Food intake				
Unchanged	111 (46)	46 (53)	27 (35)	38 (51)
More than usual	8 (3)	5 (6)	1 (1)	2 (3)
Less than usual	116 (49)	34 (39)	49 (63)	33 (45)
If less than usual, I am now taking:				
Normal food but less amount	108 (45)	31 (36)	44 (56)	33 (45)
Little solid food	3 (1)	0 (0)	3 (4)	0 (0)
Only liquids/nutritional supplements	1 (0)	0 (0)	1 (1)	0 (0)
Very little of everything	1 (0)	1 (1)	0 (0)	0 (0)
Only tube feedings/nutritional by vein	3 (1)	2 (2)	1 (1)	0 (0)
Symptoms [1]				
No problem eating	107 (45)	35 (40)	31 (40)	41 (55)
No appetite	44 (18)	15 (17)	20 (26)	9 (12)
Nausea	34 (14)	15 (17)	14 (18)	5 (7)
Constipation	7 (3)	3 (3)	3 (4)	1 (1)
Mouth sores	3 (1)	0 (0)	1 (1)	2 (3)
Things tast funny or have no taste	19 (8)	6 (7)	9 (12)	4 (5)
Problems swallowing	35 (15)	16 (18)	13 (17)	6 (8)
Pain[2]	14 (6)	4 (5)	5 (6)	5 (7)
Other[3]	13 (5)	5 (6)	5 (6)	3 (4)
Vomiting	14 (6)	4 (5)	7 (9)	3 (4)
Diarrhea	20 (8)	8 (9)	8 (10)	4 (5)
Dry mouth	11 (5)	4 (5)	2 (3)	5 (7)
Smells bother me	8 (3)	2 (2)	5 (6)	1 (1)
Feel full quickly	77 (32)	25 (29)	29 (37)	23 (31)
Total number of symptoms reported:	299	107	121	71
Activities/function				

For peer review only

Normal with no limitations	143 (60)	50 (57)	43 (55)	50 (68)
Not my normal self	70 (29)	28 (32)	21 (27)	21 (28)
Not feeling up to most things	17 (7)	6 (7)	8 (10)	3 (4)
Able to be little active	6 (3)	1 (1)	5 (6)	0 (0)
Pretty much bed ridden	1 (0)	1 (1)	0 (0)	0 (0)
**Patients with malnutrition (total sum score)**	**71 (30)**	**22 (25)**	**33 (42)**	**16 (22)**

[1] Several answers possible. (Fatigue not included in the Swedish version of the abPG-SGA (17)).

[2] No patient gave information about where they had pain.

[3] For example, throat irritation, the food does not taste like before, feeling of strangulation in end of meal, tooth problems, dumping, concern because of family problems, swelling in throat.

For peer review only

Table 3. Associations between surgical technique for esophagectomy due to cancer and nutritional status and weight loss 1 year after surgery in a Swedish population-based cohort study.

	Open esophagectomy	Minimally invasive esophagectomy	Hybrid minimally invasive esophagectomy
	(Reference)	OR (95% CI)[1]	OR (95% CI)[1]
**Malnutrition – total score from abPG-SGA**	1.0	1.86 (0.91-3.82)	0.92 (0.42-2.04)
Reduced food intake	1.0	2.87 (1.47-5.61)	1.32 (0.68-2.59)
Symptoms reducing food intake[2]	1.0	1.32 (0.67-2.61)	0.93 (0.46-1.90)
Decreased activities/function	1.0	1.81 (0.65-5.02)	0.50 (0.12-2.11)
Weight			
Weight loss 6 months after surgery			
≤10%	1.0	1.26 (0.38-4.19)	1.14 (0.32-4.02)
>10%	1.0	1.51 (0.47-4.90)	1.59 (0.47-5.40)
Weight loss 1 year after surgery			
≤10%	1.0	3.08 (0.86-10.95)	0.90 (0.31-2.61)
>10%	1.0	2.06 (0.60-7.04)	0.96 (0.36-2.57)

[1] Adjusted for age, sex, Charlson's comorbidity index, preoperative BMI, pathological tumor stage, neoadjuvant therapy, enteral/parenteral nutrition support, and postoperative complications.

[2] Nausea, diarrhoea, dry mouth, problems swallowing, feel full quickly, fatigue, pain etc.

Abbreviations: OR, Odds ratio; CI, confidence interval.

For peer review only

FIGURES

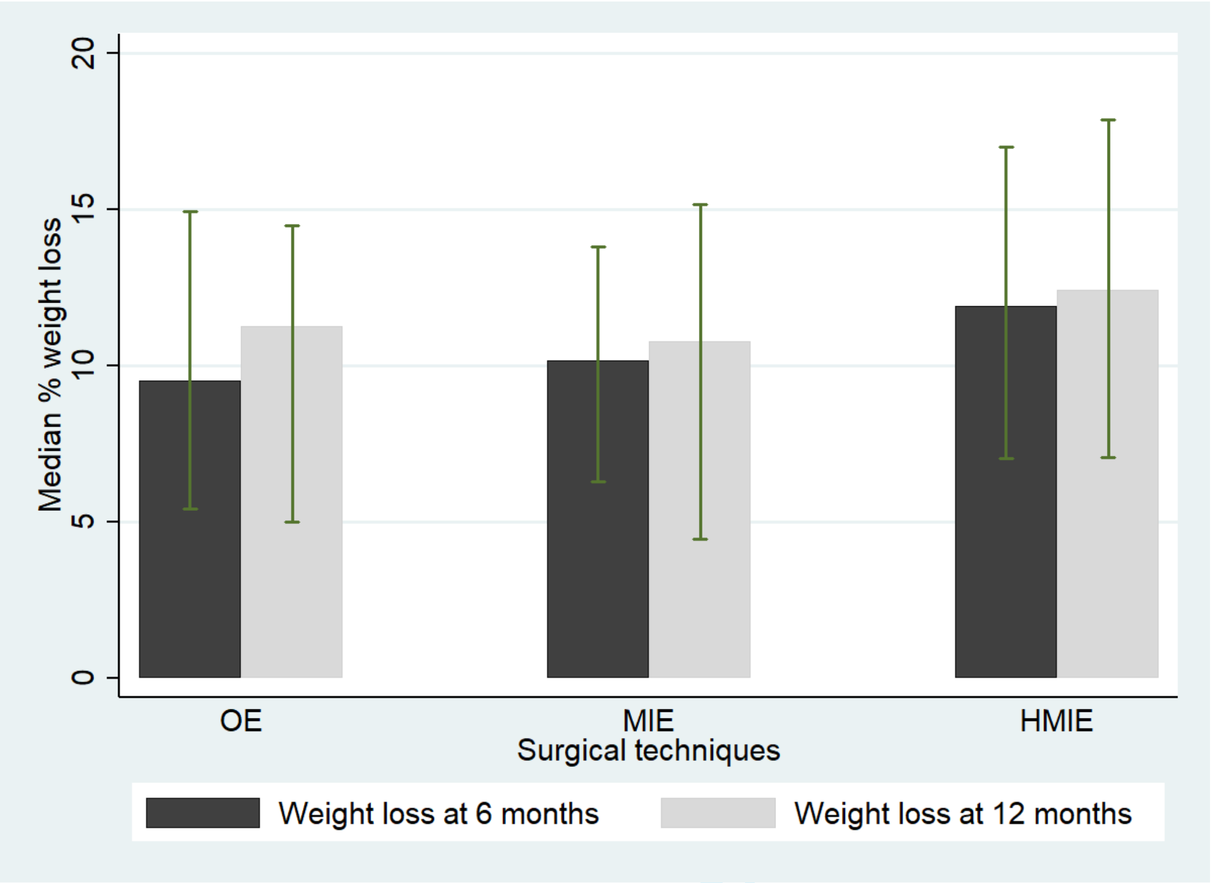

Figure 1.

APPENDIX 1

Scored Patient-generated Subjective Global Assessment (PG-SGA)

History: Boxes 1 - 4 are designed to be completed by the patient.
[Boxes 1-4 are referred to as the PG-SGA Short Form (SF)]

Patient identification information

1. Weight *(see worksheet 1)*

In summary of my current and recent weight:

I currently weigh about _____ pounds
I am about _____ feet _____ inches tall

One month ago I weighed about _____ pounds
Six month ago I weighed about _____ pounds

During the past two weeks my weight has:
☐ Decreased (1) ☐ Not changed (0) ☐ Increased (0)

Box 1 ☐

2. Food intake: As compared to my normal intake, I would rate my food intake during the past month as
- ☐ Unchanged (0)
- ☐ More than usal (0)
- ☐ Less than usal (1)

I am now taking
- ☐ *Normal food* but less than normal amount (1)
- ☐ Little solid food (2)
- ☐ Only liquids (3)
- ☐ Only nutritional supplements (3)
- ☐ Very little of anything (4)
- ☐ Only tube feedings or only nutribution by vein (0)

Box 2 ☐

3. Symptoms: I have had the following problems that have kept me from eating enough during the past two weeks (check all that apply)
- ☐ No problem eating (0)
- ☐ No appetite, just did not feel like eating (3)
- ☐ Vomiting (3)
- ☐ Nausea (1)
- ☐ Diarrhea (3)
- ☐ Constipation (1)
- ☐ Dry moth (1)
- ☐ Mouth sores (2)
- ☐ Smells bother me (1)
- ☐ Things taste funny or have no taste (1)
- ☐ Feel full quickly (1)
- ☐ Problems swallowing (2)
- ☐ Fatigue (1)
- ☐ Pain; where? (3) ____________
- ☐ Other (1) ** ____________

Examples: Depression,money, or dental problems **Box 3 ☐

4. Activities and function:
Over the past month, I would generally rate my activity as:
- ☐ Normal with no limitations (0)
- ☐ Not my normal self, but able to be up and about with fairly normal activities (1)
- ☐ Not feeling up to most things, but in bed or chair less than half the day (2)
- ☐ Able to be little activity and spend most of the day in bed or chair (3)
- ☐ Pretty much bed ridden, rarely out of bed (3)

Box 4 ☐

The remainder of this form is to be completed by your doctor, nurse, dietitian, or therapist. Thank you.

Additive score of boxes 1-4 ☐ **A**

©FD Ottery 2005, 2006, 2015 v3.22.15
email: faithotterymdphd@aol.com or info@pt-global.org

1

STROBE Statement—Checklist of items that should be included in reports of ***cohort studies***

	Item No	Recommendation	Page No
**Title and abstract**	1	(*a*) Indicate the study's design with a commonly used term in the title or the abstract	1
		(*b*) Provide in the abstract an informative and balanced summary of what was done and what was found	2
**Introduction**			
Background/rationale	2	Explain the scientific background and rationale for the investigation being reported	4
Objectives	3	State specific objectives, including any prespecified hypotheses	4
**Methods**			
Study design	4	Present key elements of study design early in the paper	5
Setting	5	Describe the setting, locations, and relevant dates, including periods of recruitment, exposure, follow-up, and data collection	5
Participants	6	(*a*) Give the eligibility criteria, and the sources and methods of selection of participants. Describe methods of follow-up	5
		(*b*) For matched studies, give matching criteria and number of exposed and unexposed	
Variables	7	Clearly define all outcomes, exposures, predictors, potential confounders, and effect modifiers. Give diagnostic criteria, if applicable	5-7
Data sources/ measurement	8*	For each variable of interest, give sources of data and details of methods of assessment (measurement). Describe comparability of assessment methods if there is more than one group	5-7
Bias	9	Describe any efforts to address potential sources of bias	7-8,11
Study size	10	Explain how the study size was arrived at	9
Quantitative variables	11	Explain how quantitative variables were handled in the analyses. If applicable, describe which groupings were chosen and why	7-8
Statistical methods	12	(*a*) Describe all statistical methods, including those used to control for confounding	7-8
		(*b*) Describe any methods used to examine subgroups and interactions	N/A
		(*c*) Explain how missing data were addressed	9
		(*d*) If applicable, explain how loss to follow-up was addressed	
		(*e*) Describe any sensitivity analyses	
**Results**			
Participants	13*	(a) Report numbers of individuals at each stage of study—eg numbers potentially eligible, examined for eligibility, confirmed eligible, included in the study, completing follow-up, and analysed	9
		(b) Give reasons for non-participation at each stage	9
		(c) Consider use of a flow diagram	N/A
Descriptive data	14*	(a) Give characteristics of study participants (eg demographic, clinical, social) and information on exposures and potential confounders	9
		(b) Indicate number of participants with missing data for each variable of interest	N/A
		(c) Summarise follow-up time (eg, average and total amount)	N/A
Outcome data	15*	Report numbers of outcome events or summary measures over time	9-10

For peer review only

For peer review only - http://bmjopen.bmj.com/site/about/guidelines.xhtml

Main results	16	(*a*) Give unadjusted estimates and, if applicable, confounder-adjusted estimates and their precision (eg, 95% confidence interval). Make clear which confounders were adjusted for and why they were included	9-10
		(*b*) Report category boundaries when continuous variables were categorized	19
		(*c*) If relevant, consider translating estimates of relative risk into absolute risk for a meaningful time period	N/A
Other analyses	17	Report other analyses done—eg analyses of subgroups and interactions, and sensitivity analyses	10
**Discussion**			
Key results	18	Summarise key results with reference to study objectives	11
Limitations	19	Discuss limitations of the study, taking into account sources of potential bias or imprecision. Discuss both direction and magnitude of any potential bias	11
Interpretation	20	Give a cautious overall interpretation of results considering objectives, limitations, multiplicity of analyses, results from similar studies, and other relevant evidence	13
Generalisability	21	Discuss the generalisability (external validity) of the study results	11
**Other information**			
Funding	22	Give the source of funding and the role of the funders for the present study and, if applicable, for the original study on which the present article is based	14

*Give information separately for exposed and unexposed groups.

Note: An Explanation and Elaboration article discusses each checklist item and gives methodological background and published examples of transparent reporting. The STROBE checklist is best used in conjunction with this article (freely available on the Web sites of PLoS Medicine at http://www.plosmedicine.org/, Annals of Internal Medicine at http://www.annals.org/, and Epidemiology at http://www.epidem.com/). Information on the STROBE Initiative is available at http://www.strobe-statement.org.

For peer review only

BMJ Open

Minimally invasive surgical techniques for esophageal cancer and nutritional recovery – a prospective population-based cohort study.

Journal:	*BMJ Open*
Manuscript ID	bmjopen-2021-058763.R1
Article Type:	Original research
Date Submitted by the Author:	30-Mar-2022
Complete List of Authors:	Kauppila, Joonas; Karolinska University Hospital, Department of Molecular medicine and Surgery; University of Oulu, Department of Surgery<br>Rosenlund, Helen; Karolinska University Hospital, Department of Molecular medicine and Surgery; Danderyds Sjukhus AB, Department of Orthopaedics<br>Klevebro, Fredrik; Karolinska Institutet, Surgical Care Science<br>Johar, Asif; Karolinska Institutet, Department of Molecular medicine and Surgery<br>Anandavadivelan, Poorna; Karolinska Institutet, Department of Molecular medicine and Surgery<br>Mälberg, Kalle; Karolinska Institutet, Upper Gastrointestinal Research, Department of Molecular Medicine and Surgery<br>Lagergren, Pernilla; Karolinska Institute, Department of Molecular Medicine and Surgery
**Primary Subject Heading**:	Surgery
Secondary Subject Heading:	Nutrition and metabolism
Keywords:	Oesophageal disease < GASTROENTEROLOGY, SURGERY, NUTRITION & DIETETICS

SCHOLARONE™
Manuscripts

I, the Submitting Author has the right to grant and does grant on behalf of all authors of the Work (as defined in the below author licence), an exclusive licence and/or a non-exclusive licence for contributions from authors who are: i) UK Crown employees; ii) where BMJ has agreed a CC-BY licence shall apply, and/or iii) in accordance with the terms applicable for US Federal Government officers or employees acting as part of their official duties; on a worldwide, perpetual, irrevocable, royalty-free basis to BMJ Publishing Group Ltd ("BMJ") its licensees and where the relevant Journal is co-owned by BMJ to the co-owners of the Journal, to publish the Work in this journal and any other BMJ products and to exploit all rights, as set out in our licence.

The Submitting Author accepts and understands that any supply made under these terms is made by BMJ to the Submitting Author unless you are acting as an employee on behalf of your employer or a postgraduate student of an affiliated institution which is paying any applicable article publishing charge ("APC") for Open Access articles. Where the Submitting Author wishes to make the Work available on an Open Access basis (and intends to pay the relevant APC), the terms of reuse of such Open Access shall be governed by a Creative Commons licence – details of these licences and which Creative Commons licence will apply to this Work are set out in our licence referred to above.

Other than as permitted in any relevant BMJ Author's Self Archiving Policies, I confirm this Work has not been accepted for publication elsewhere, is not being considered for publication elsewhere and does not duplicate material already published. I confirm all authors consent to publication of this Work and authorise the granting of this licence.

For peer review only

Minimally invasive surgical techniques for esophageal cancer and nutritional recovery – a prospective population-based cohort study.

Joonas H. Kauppila PhD[1,2], Helen Rosenlund PhD[1,3], Fredrik Klevebro PhD[1], Asif Johar MSc[1], Poorna Anandavadivelan PhD[1], Kalle Mälberg MSc[1], Pernilla Lagergren PhD[1,4]

[1] Surgical Care Sciences, Department of Molecular medicine and Surgery, Karolinska Institutet, Karolinska University Hospital 171 76 Stockholm, Sweden.

[2] Department of Surgery, Oulu University Hospital, University of Oulu, Finland

[3] Clinical Nutrition and Dietetics, Department of Orthopaedics, Danderyd Hospital, Stockholm, Sweden.

[4] Department of Surgery & Cancer, Imperial College London, United Kingdom

Corresponding author and requests for reprints:

Joonas H. Kauppila, Surgical Care Science, Department of Molecular medicine and Surgery, Karolinska Institutet, Karolinska University Hospital, Retzius väg 13A, 171 77 Stockholm, Sweden; E-mail: joonas.kauppila@ki.se

Running title: Surgical techniques and nutritional recovery.

Funding: The Swedish Cancer Society, the Stockholm County Council, and the Cancer Research Funds of Radiumhemmet.

Conflict of interest: All authors declare that they have no conflict of interest in relation to this manuscript.

For peer review only

ABSTRACT (Word count 283)

Objectives: To explore whether the minimally invasive (MIE), or hybrid minimally invasive esophagectomy (HMIE) are associated with better nutritional status and less weight loss 1 year after surgery, compared with open esophagectomy (OE).

Design: Prospective cohort study

Setting: All patients undergoing esophagectomy for cancer in Sweden during 2013-2018.

Participants: A total of 424 patients alive at 1 year after surgery were eligible, and 281 completed the 1-year assessment. Of these, 239 had complete clinical data and were included in the analysis.

Primary and secondary outcome measure: The primary outcome was nutritional status at 1 year after surgery, assessed using the abPG-SGA questionnaire. The secondary outcomes included postoperative weight loss at 6 months and 1 year after surgery.

Results: Of the included patients, 78 underwent MIE, 74 HMIE while 87 patients underwent OE. The MIE group had the highest prevalence of malnutrition (42% vs. 22% after HMIE vs. 25% after OE), reduced food intake (63% vs 45% after HMIE vs. 39% after OE), symptoms reducing food intake (60% vs. 45% after HMIE vs. 60% after OE) and abnormal activities/function (45% vs. 32% after HMIE vs 43% after OE). After adjustment for confounders, MIE was associated with a statistically significant increased risk of reduced food intake 1 year after surgery (OR 2.87, 95% CI 1.47-5.61), compared to OE. Other outcomes were not statistically significantly different between the groups. No statistically significant associations were observed between surgical techniques and weight loss up to 1 year after surgery.

Conclusions: MIE was statistically significantly associated with reduced food intake 1 year after surgery. However, no differences were observed in weight loss between the surgical

For peer review only

techniques. Further studies on nutritional impact of surgical techniques in esophageal cancer are needed.

Keywords: esophageal cancer; minimally invasive surgery; nutrition

Strengths and limitations of this study:

- The strength of this study is the prospective and population-based study design with longitudinal collection of data according to a predefined study protocol.

- The relatively high inclusion rate and complete data are strengths.

- The abPG-SGA questionnaire is validated and commonly used to measure malnutrition in oncology patients.

- A weakness of this study was that all patients who died during 1 year after esophagectomy were not eligible.

For peer review only

INTRODUCTION

The mainstay of curative treatment for esophageal cancer is surgery which carries a high risk of postoperative complications and mortality (1). The surgery is often combined with preoperative neoadjuvant therapy (2). The traditional open esophagetomy (OE) with abdominal and thoracic incisions is being replaced by minimally invasive esophagectomy (MIE), performed using thoracoscopy and laparoscopy (3). MIE is associated with lower risk of postoperative complications, especially pulmonary, lower in-hospital mortality, shorter length of hospital stay, and lower 90-day mortality, compared to OE (4-7). MIE has also been associated with improved health-related quality of life (HRQL) (8, 9). Hybrid minimally invasive esophagectomy (HMIE) applies open surgery in combination with laparoscopic or thoracoscopic technique, e.g. a thoracotomy and laparoscopy. HMIE is associated less postoperative mortality and better HRQL, compared with OE (10).

Esophageal cancer is associated with significant nutritional problems and weight loss before and after treatment (11-13). Patients lose between 5-12% of their preoperative weight during the first 6 months after esophagectomy and a majority have lost more than 10% of their body weight 12 months postoperatively (14). However, the effect of MIE on nutritional recovery remains unclear. It was hypothesized that MIE could improve nutritional recovery after esophagectomy, compared to OE.

The aim of this study was to investigate the impact of surgical technique, used for esophagectomy for cancer, on nutritional status and weight loss up to 1 year after surgery in a population-based and nationwide setting.

For peer review only

MATERIALS AND METHODS

Study design

This project was based on the nationwide and prospective cohort entitled Oesophageal Surgery on Cancer patients–Adaptation and Recovery (OSCAR) (15). Patients who underwent surgery for esophageal cancer in Sweden from 1st January 2013 and onwards were included and first inclusion started 1st January 2014. Eligible patients were identified from pathology units at eight treating hospitals in Sweden. An invitation letter was sent and within 1 week patients were contacted by telephone to get information regarding the project and to give their consent to participate. Arrangements for the first assessment were also made. A research nurse visited the patients in their homes for collection of patient-reported outcomes, objective measures of body weight and body composition. The OSCAR study was approved by the Regional Ethical Review Board in Stockholm, Sweden and all participants provided written informed consent.

Clinical data at time of surgery was collected from medical records, which were reviewed according to a predefined study protocol to ensure uniformity: 1) Patient characteristics including age, sex, comorbidities, and weight at time of surgery; 2) Tumor histology, site/stage; 3) Treatment, including neoadjuvant or adjuvant oncological treatment, and surgical approach; 4) Postoperative complications classified according to the Clavien-Dindo scoring system (16).

view only

Exposures

The following three types of surgery were used as exposures: 1) open transthoracic or transhiatal esophagectomy (OE; reference group), 2) minimally invasive esophagectomy (thoracoscopic-laparoscopic) (MIE), and 3) hybrid minimally invasive esophagectomy

(thoracoscopic/open abdomen or laparoscopic/open chest) (HMIE). Data on surgical technique was obtained from the medical records.

Outcomes

The primary outcome was malnutrition measured by the abPG-SGA (abbreviated patient-generated subjective global assessment) questionnaire (Supplementary Figure 1), that was completed by the patient at one time-point 1 year after surgery. This is a screening tool to detect patients at risk of malnutrition and is recommended for use in oncology patients (17, 18). The abPG-SGA is an abbreviated version of the PG-SGA questionnaire and has been found valid to detect malnutrition when used in an outpatient oncology clinic (19).

The abPG-SGA contains four sections which concern body weight, food intake, symptoms reducing food intake, and activities/function. The first section about body weight asks for the patient's current weight and height, weight at 1 and 6 months ago, and if the patient's weight has "decreased"/"increased"/"has been stable" during the last two weeks. The second section concerns food intake where the patient compares his/her food intake to their normal intake as "unchanged"/"more than usual"/"less than usual". The patient is then asked to relate his/her food intake to the habitual food intake prior to cancer symptoms/diagnosis (in the original questionnaire patients related food intake to 1 month ago). If the answer was "less than usual" the patient responded to the follow-up question: What type of food do you eat? with the following response alternatives "normal food but less than normal amount"/"little solid food"/"only liquids"/"only nutritional supplements"/"very little of anything"/"only tube feeding or parenteral nutrition". Section three covers symptoms that have kept the patient from eating enough during the past two weeks (several answers possible): "no problem"/"no appetite, just did not feel like eating"/"nausea"/"vomiting"/"constipation"/

For peer review only

"diarrhoea"/"mouth sores"/"dry mouth"/"things taste funny or have no taste"/"smells bother me"/"problems swallowing"/"feel full quickly"/"pain"/"other". The forth section concerns activities/function and asks the patients to rate their general activity over the past months: "normal"/"not my normal self, but have fairly normal activities"/"not feeling up to things, in bed/chair half the day"/"able to be little active and spend most of the day in bed/chair"/"rarely out of bed". The score from each part totals up a sum that determines the patient´s nutritional status. A higher score indicates more severe malnutrition (17, 19).

The secondary outcomes were postoperative weight loss between 1) time of surgery and 6 months postoperatively and 2) time of surgery and 1 year after surgery. The weight at surgery was collected from medical records, weight at 6 months was reported by the patient in the abPG-SGA questionnaire, and weight at 1 year was measured by the research nurse. Weight loss was calculated by comparing weight measures at surgery and weight at 6 months and at 1 year.

Statistical analysis

Logistic regression models were used to analyze the associations between surgical techniques (OE [reference group], MIE, HMIE) and malnutrition (yes, no) and weight loss at 6 months and at 1 year postoperatively. Weight loss was categorized as 1) No weight loss/weight gain, 2) ≤10% weight loss, 3) >10% weight loss. Results were presented as odds ratios (OR) with 95% confidence intervals (CI), adjusted for potential confounders: age (longitudinal), sex (male, female), Charlson's comorbidity index (0, 1, ≥2), preoperative body mass index (BMI) (<25, ≥25), pathological tumor stage (0-I, II, III-IV), neoadjuvant therapy (yes, no), enteral/parenteral nutrition support during at least 1 week postoperatively (yes, no), and postoperative complications (Clavien-Dindo score [CDS]; low grade [0-II], high grade [III-

For peer review only

IV]). Furthermore, an explanatory analysis was conducted to further adjust for anastomotic leak defined as clinically significant or radiologically confirmed anastomotic leak (yes, no), intensive care unit stay (continuous) and hospital stay (continuous). Weight loss was also presented as median weight loss percentage, combined with the inter quartile range for each group. In the analyses of the abPG-SGA questionnaire, a summarized score of ≥6 was considered as malnutrition. For body weight, food intake, symptoms reducing food intake, and activities/function a score of ≥2 was considered as a lower/worse condition in each group, with a reference score of <2. A P-value <0.05 was considered statistically significant.

Patient and public involvement

The Surgical Care Science research partnership group including patients with esophageal cancer were involved in the planning of OSCAR cohort study. The partnership group patients were involved in discussions around the study questions, as well as on the potential outcome measures collected during the study. The partnership group were not involved in recruitment of participants, but have been involved in the dissemination of research findings and increasing public awareness of esophageal cancer. Regarding the present study, the research partnership group patients suggested nutritional as an important outcome requiring more studies. Findings from this study will be communicated to patients and healthcare professionals through conferences and patient meetings.

For peer review only

RESULTS

Participants

Between January 2013 and April 2018, 675 patients underwent esophageal cancer surgery in Sweden. Of these, 511 (76%) survived for at least 1 year, 85 were not reachable, and 2 were excluded because of cognitive impairment, leaving 424 patients eligible for inclusion. Of these, 281 (66%) patients completed the 1-year assessment and clinical data were available for 247 (58%) patients. In total, 239 patients were included in the analyses. A comparison of study population and a population-based nationwide study of patients undergoing esophagectomy in Sweden during the same period to examine the potential selection bias suggested no major difference in distribution of different surgical approaches in this study compared to population (Supplementary Table 1).

Patient characteristics

Patients were evenly distributed in the 3 surgical groups (OE 36%, MIE 33%, and HMIE 31%) (Table 1). The mean age was 66.3 (±8.7) years at time of surgery and most patients were male (87%). The distribution of preoperative BMI and weight loss were similar across the three groups. Preoperative neoadjuvant therapy was most often used in the HMIE group (84%), while a higher pathological tumor stage was more frequently seen in the MIE group (37%), compared to the other groups. Almost all patients had a gastric conduit, while few patients in the OE group had an esophagojejunostomy. A neck anastomosis was more common in the MIE group, compared to the OE and HMIE groups. Patients who underwent HMIE less often received enteral/parenteral nutrition for at least 1 week, compared to the other groups. Patients with jejunostomy received enteral feeding for 14 days in median.

For peer review only

Nutritional status

One third (30%) of all patients had malnutrition 1 year postoperatively, based on the abPG-SGA (Table 2), and it was highest in the MIE group (42%), compared to the OE (25%) and the HMIE (22%) groups. The MIE group had the highest prevalence of reduced food intake (63% vs 45% after HMIE vs. 39% after OE), symptoms reducing food intake (60% vs. 45% after HMIE vs. 60% after OE). The number of reported symptoms reducing food intake (each symptom counted once for each patient, one patient could have multiple symptoms) in the MIE group was higher in the MIE group (121), compared to HMIE (71) and OE (107). Furthermore, abnormal activities/function were more common after MIE (45% vs. 32% after HMIE vs 43% after OE). In multivariable analysis, the point estimate for the risk of malnutrition was high after MIE, compared to OE (OR_{adj} 1.86, 95% CI 0.91-3.82), but this difference was statistically non-significant. A statistically significant association was observed between MIE and reduced food intake at 1 year after surgery (OR_{adj} 2.87, 95% CI 1.47-5.61), compared to OE (Table 3). No associations were observed between surgical technique and symptoms reducing food intake or activities/function.

In a sensitivity analysis the level of anastomosis was included in the model, but the results remained the same, except that the point estimate of reduced food intake after MIE was increased to 3.13 (95% CI 1.53-6.39). Furthermore, in an attempt to minimize the risk that patients in the OE group reconstructed with jejunal conduit affected the outcomes, these 8 patients were excluded from the analyses, but the results remain unchanged. Lastly, adjustment for anastomotic leak, intensive care unit stay and hospital stay in the explanatory analysis did not change the point estimates compared to the main analysis (Supplementary Table 2).

For peer review only

Weight loss

The patients lost most weight during the first 6 months postoperatively in all groups and it was most pronounced after HMIE, compared to both OE and MIE. A similar pattern was observed for weight loss at 1 year after surgery (Figure 1). Surgical technique was not associated with weight loss at 6 months or 1 year after surgery (Table 3). Additional adjustment for anastomotic leak, intensive care unit stay and hospital stay did not change the estimates (Supplementary Table 2).

For peer review only

DISCUSSION

The results of this population-based nationwide cohort study suggest that patients operated with MIE and HMIE had no increased risk in malnutrition compared to OE, but those operated with MIE were more likely to have reduced food intake 1 year postoperatively, compared to OE. No association between surgical technique and weight loss up to 1 year postoperatively was present.

The strength of this study is the prospective and population-based study design with longitudinal collection of data. Clinical data at time of surgery was collected from medical records and reviewed according to a predefined study protocol to ensure consistency and uniformity of the data. The cohort had relatively high inclusion rate, and completeness of the abPG-SGA questionnaire in survivors 1 year postoperatively was very high. The abPG-SGA questionnaire is a validated and commonly-used instrument that has been reported to be a valid tool to measure malnutrition in oncology patients (19). The results may be generalizable to hospitals in Western countries where these surgical techniques are used. Missing data is in general low when collected by a research nurse in patients' homes. Weaknessess of this study include that all patients who died during 1 year after esophagectomy were not eligible, and that there is potential selection bias at the inclusion time of 1 year after surgery despite the distributions of surgical approaches being similar in this study and the population. Moreover, the patients´ self-reported weight measure could be biased, although this is assumed to be a minor problem since keeping track of weight is a central concern among these patients. In the section about food intake in the abPG-SGA, the nurse asked these patients to relate their actual food intake to their habitual eating prior to diagnosis, instead of 1 month ago, to make it more suitable for this patient group. This may give a more correct picture of the nutritional

For peer review only

status in this group since comparing to 1 month ago would not have shown any difference because their eating problems started at time of surgery or earlier.

Previous studies comparing malnutrition between MIE and OE are sparse, as very few have looked at malnutrition in the long term, and none using the abPG-SGA questionnaire. The previous studies assessing malnutrition have used weight loss or BMI as a proxy for nutritional status(20, 21) or laboratory values (6, 22). In an Italian case-control study, serum albumin levels were higher at day 1, 3, and 8 in patients undergoing HMIE, compared to OE.(22) An American study suggested earlier oral intake in patients who underwent MIE, compared to OE(6). The results from the current study suggest that MIE has a greater negative effect on the patients´ food intake, compared to OE, which is the opposite of what we hypothesized *a priori*. However, there was no significant difference in malnutrition, or other nutritional symptoms between the surgical techniques. Based on previous research, MIE should lead to less postoperative complications and shorter length of hospital stay, compared to OE (5-7). In the present study, major complications were similar between the groups and likely not a relevant factor in food intake symptoms. Furthermore, an additional adjustment for anastomotic leak, intensive care unit stay, and hospital stay in the explanatory analysis did not result in major changes in the point estimates compared to the main analysis. On the other hand, neck anastomosis is known to be associated with more anastomotic strictures, compared to chest anastomosis, which might affect the food intake(23). As the MIE group had more neck anastomoses than HMIE and OE groups, a sensitivity analysis adjusted for anastomotic location was conducted but the results were no different from the main analysis. The conduit used for reconstruction might also affect food intake, but excluding jejunal interposition grafts did not change the results. Anastomotic techniques (i.e., handsewn vs circular stapled vs. linear stapled) might also differ between MIE, HMIE and OE. For example, end-to-side

For peer review only

stapled and hand-sewn, as well as linear stapled side-to-side anastomoses have been described in Sweden(24, 25). However, these differences remain speculative, as this data was not available, and further studies are needed. Lastly, early oral feeding is central to any Enhanced Recovery After Surgery (ERAS) programs, (26) and could impact the long-term nutritional recovery of these patients. However, participation in ERAS programs was not available in the dataset. As esophageal cancer strongly affects the patients´ food intake before and after surgery, the associated weight loss is often substantial(27). Some previous studies have investigated how surgical technique affects patients´ weight in the long-term. A Swedish clinical study observed no difference in weight loss during the first postoperative year in patients who underwent MIE, compared to OE (20). Our results suggested a higher weight loss in the HMIE group, both at 6 months and 1 year postoperatively, but this difference was non-significant in the adjusted analyses. In addition to no nutritional differences between the surgical methods, the results could be explained by too small number of patients in the analysis. However, the weight loss in the OE group was increasing up to 1 year after surgery, and studies with longer-term follow-up might be warranted.

Even though there were no major differences in malnutrition between minimally invasive and open esophagectomy, a striking finding of this study is that one third (30%) of all included patients were malnourished at 1 year after surgery. This highlights the importance of nutritional support and long-term follow-up from the health care for patients who undergo esophagectomy. Since esophagectomy results in a permanent anatomical change of the upper gastrointestinal tract, besides nutritional strategies in the immediate postoperative phase such as early enteral feeding, nutritional interventions for postoperative complications, attention to long-term nutritional intake and status are also of high importance(27). While surgical technique might not be of high importance, the impact of anastomotic technique on nutrition

For peer review only

in esophageal cancer patients should be further evaluated. With improved knowledge, the utility of long-term dietitian support in different patient groups could be clarified.

In conclusion, this population-based prospective cohort study suggests that while there was no increased risk of malnutrition between minimally invasive compared to open esophagectomy, MIE is associated to reduced food intake 1 year postoperatively. No significant differences were present between surgical technique and weight loss. Further studies on nutritional impact of surgical techniques in esophageal cancer are needed.

ACKNOWLEDGEMENTS

We thank all study participants and the members of the Surgical Care Science patient research partnership group for their contribution to the OSCAR study.

Contributorship statement

JHK, HR, FK, PA, and PL contributed with input in designing the study. Data collection and assembly of data were performed by KM and PL. Data analysis was conducted by AJ. All authors (JHK, HR, FK, AJ, PA, KM, and PL) contributed in interpreting the results, manuscript writing and approval of the final version of the manuscript.

Competing interests

The authors state no conflicts of interest.

Funding statement

For peer review only

We also would like to thank the Swedish Cancer Society (grant number 180685), the Cancer Research Funds of Radiumhemmet (grant number 171103), and the Stockholm County Council (grant number LS 2018–1157) for financial support. Pernilla Lagergren is supported by the NIHR Imperial Biomedical Research Centre (BRC) for her position at Imperial College London, London, United Kingdom. The funding sources had no role in the design and conduct of the data collection, management, analysis and interpretation of the data or preparation review or approval of the manuscript.

Data Sharing Statement

All data underlying the findings are presented in the manuscript. The datasets generated and/or analyzed in the current study will not be publicly available due to the ethical review act but will be available from the principal investigators of the study on reasonable request.

For peer review only

REFERENCES

1. Markar SR, Karthikesalingam A, Low DE. Outcomes assessment of the surgical management of esophageal cancer in younger and older patients. Ann Thorac Surg. 2012;94(5):1652-8.
2. Lagergren J, Smyth E, Cunningham D, Lagergren P. Oesophageal cancer. Lancet. 2017;390(10110):2383-96.
3. Lazzarino AI, Nagpal K, Bottle A, Faiz O, Moorthy K, Aylin P. Open versus minimally invasive esophagectomy: trends of utilization and associated outcomes in England. Ann Surg. 2010;252(2):292-8.
4. Briez N, Piessen G, Torres F, Lebuffe G, Triboulet JP, Mariette C. Effects of hybrid minimally invasive oesophagectomy on major postoperative pulmonary complications. Br J Surg. 2012;99(11):1547-53.
5. Yibulayin W, Abulizi S, Lv H, Sun W. Minimally invasive oesophagectomy versus open esophagectomy for resectable esophageal cancer: a meta-analysis. World J Surg Oncol. 2016;14(1):304.
6. Tapias LF, Mathisen DJ, Wright CD, Wain JC, Gaissert HA, Muniappan A, et al. Outcomes With Open and Minimally Invasive Ivor Lewis Esophagectomy After Neoadjuvant Therapy. Ann Thorac Surg. 2016;101(3):1097-103.
7. Kauppila JH, Helminen O, Kyto V, Gunn J, Lagergren J, Sihvo E. Short-Term Outcomes Following Minimally Invasive and Open Esophagectomy: A Population-Based Study from Finland and Sweden. Ann Surg Oncol. 2018;25(1):326-32.
8. Kauppila JH, Xie S, Johar A, Markar SR, Lagergren P. Meta-analysis of health-related quality of life after minimally invasive versus open oesophagectomy for oesophageal cancer. Br J Surg. 2017;104(9):1131-40.
9. Maas KW, Cuesta MA, van Berge Henegouwen MI, Roig J, Bonavina L, Rosman C, et al. Quality of Life and Late Complications After Minimally Invasive Compared to Open Esophagectomy: Results of a Randomized Trial. World J Surg. 2015;39(8):1986-93.
10. Mariette C, Markar SR, Dabakuyo-Yonli TS, Meunier B, Pezet D, Collet D, et al. Hybrid Minimally Invasive Esophagectomy for Esophageal Cancer. N Engl J Med. 2019;380(2):152-62.
11. Haverkort EB, Binnekade JM, Busch OR, van Berge Henegouwen MI, de Haan RJ, Gouma DJ. Presence and persistence of nutrition-related symptoms during the first year following esophagectomy with gastric tube reconstruction in clinically disease-free patients. World J Surg. 2010;34(12):2844-52.
12. Anandavadivelan P, Lagergren P. Cachexia in patients with oesophageal cancer. Nat Rev Clin Oncol. 2016;13(3):185-98.
13. Bozzetti F. Nutritional support in patients with oesophageal cancer. Support Care Cancer. 2010;18 Suppl 2:S41-50.
14. Baker M, Halliday V, Williams RN, Bowrey DJ. A systematic review of the nutritional consequences of esophagectomy. Clin Nutr. 2016;35(5):987-94.
15. Schandl A, Johar A, Anandavadivelan P, Vikstrom K, Malberg K, Lagergren P. Patient-reported outcomes 1 year after oesophageal cancer surgery. Acta Oncol. 2020:1-7.
16. Clavien PA, Barkun J, de Oliveira ML, Vauthey JN, Dindo D, Schulick RD, et al. The Clavien-Dindo classification of surgical complications: five-year experience. Ann Surg. 2009;250(2):187-96.

For peer review only

17. Persson C, Sjoden PO, Glimelius B. The Swedish version of the patient-generated subjective global assessment of nutritional status: gastrointestinal vs urological cancers. Clin Nutr. 1999;18(2):71-7.
18. Bauer J, Capra S, Ferguson M. Use of the scored Patient-Generated Subjective Global Assessment (PG-SGA) as a nutrition assessment tool in patients with cancer. Eur J Clin Nutr. 2002;56(8):779-85.
19. Gabrielson DK, Scaffidi D, Leung E, Stoyanoff L, Robinson J, Nisenbaum R, et al. Use of an abridged scored Patient-Generated Subjective Global Assessment (abPG-SGA) as a nutritional screening tool for cancer patients in an outpatient setting. Nutr Cancer. 2013;65(2):234-9.
20. Ericson J, Lundell L, Klevebro F, Kamiya S, Nilsson M, Rouvelas I. Long-term weight development after esophagectomy for cancer-comparison between open Ivor-Lewis and minimally invasive surgical approaches. Dis Esophagus. 2018.
21. Hynes O, Anandavadivelan P, Gossage J, Johar AM, Lagergren J, Lagergren P. The impact of pre- and post-operative weight loss and body mass index on prognosis in patients with oesophageal cancer. Eur J Surg Oncol. 2017;43(8):1559-65.
22. Scarpa M, Cavallin F, Saadeh LM, Pinto E, Alfieri R, Cagol M, et al. Hybrid minimally invasive esophagectomy for cancer: impact on postoperative inflammatory and nutritional status. Dis Esophagus. 2016;29(8):1064-70.
23. van Workum F, van der Maas J, van den Wildenberg FJ, Polat F, Kouwenhoven EA, van Det MJ, et al. Improved Functional Results After Minimally Invasive Esophagectomy: Intrathoracic Versus Cervical Anastomosis. Ann Thorac Surg. 2017;103(1):267-73.
24. Rutegard M, Lagergren J, Rouvelas I, Lindblad M, Blazeby JM, Lagergren P. Population-based study of surgical factors in relation to health-related quality of life after oesophageal cancer resection. Br J Surg. 2008;95(5):592-601.
25. Nilsson M, Kamiya S, Lindblad M, Rouvelas I. Implementation of minimally invasive esophagectomy in a tertiary referral center for esophageal cancer. J Thorac Dis. 2017;9(Suppl 8):S817-S25.
26. Weimann A, Braga M, Carli F, Higashiguchi T, Hubner M, Klek S, et al. ESPEN guideline: Clinical nutrition in surgery. Clin Nutr. 2017;36(3):623-50.
27. Steenhagen E, van Vulpen JK, van Hillegersberg R, May AM, Siersema PD. Nutrition in peri-operative esophageal cancer management. Expert Rev Gastroenterol Hepatol. 2017;11(7):663-72.

For peer review only

FIGURE LEGENDS

Figure 1. Median (%) weight loss at 6 months and at 1 year after surgery in patients who underwent esophagectomy with different surgical techniques; OE (open esophagectomy), MIE (minimally invasive esophagectomy), HMIE (hybrid minimally invasive esophagectomy).

For peer review only

Table 1. Distribution of patient characteristics in relation to the surgical technique used for esophagectomy for cancer in a Swedish population-based cohort study.

	All	Open esophagectomy (OE)	Minimally invasive esophagectomy (MIE)	Hybrid minimally invasive esophagectomy (HMIE)
	Number (%)			
Number of patients	239 (100)	87 (36)	78 (33)	74 (31)
Sex (male)	207 (87)	69 (79)	70 (90)	68 (92)
**At time of surgery**				
Age (mean + 1 SD)	66.3 + 8.7	65.8 + 8.4	66.2 + 9.8	66.9 + 7.9
Preoperative BMI (mean + 1 SD)				
<25	36 (15)	15 (17)	12 (15)	9 (12)
≥25	203 (63)	72 (83)	66 (85)	65 (88)
Preoperative weight loss				
No weight loss/weight gain	23 (10)	10 (11)	4 (5)	9 (12)
<10%	79 (33)	26 (30)	32 (41)	21 (28)
≥10%	137 (57)	51 (59)	42 (54)	44 (59)
Preoperative neoadjuvant treatment	188 (79)	68 (78)	58 (74)	62 (84)
Pathological tumor stage				
0-I	79 (33)	32 (37)	16 (21)	31 (42)
II	80 (33)	26 (30)	33 (42)	21 (28)
III-IV	80 (33)	29 (33)	29 (37)	22 (30)
Organ substitute				
Gastric tube	219 (92)	73 (84)	74 (95)	72 (97)
Esophagojejunostomy	10 (4)	8 (9)	1 (1)	1 (1)
Colonic interposition	2 (1)	1 (1)	1 (1)	0 (0)
Level of anastomosis				
Neck	38 (16)	9 (10)	20 (26)	9 (12)
Thorax	187 (78)	70 (80)	54 (69)	63 (85)
Abdomen	6 (3)	5 (6)	0 (0)	1 (1)
Charlson's comorbidity index				
0	116 (54)	43 (49)	37 (47)	36 (49)
1	77 (28)	27 (31)	23 (29)	27 (36)
≥2	46 (18)	17 (20)	18 (23)	11 (15)
Enteral/parenteral nutrition	175 (73)	68 (78)	60 (77)	47 (64)
Postoperative complications				
Low grade (CDS 0-II)	144 (60)	51 (59)	44 (56)	49 (66)
High grade (CDS III-IV)	95 (40)	36 (41)	34 (44)	25 (34)
Anastomotic leak	41 (17)	9 (22)	21 (51)	11 (27)
ICU stay (median, [IQR])	2 [1-5]	3 [1-6]	3 [1-6]	2 [1-3]
Hospital stay (median, [IQR])	16 [12-25]	16 [13-26]	16.5 [12-29]	15 [11-20]

For peer review only

SD=Standard Deviation, BMI=Body Mass Index, CDS=Clavien-Dindo Score.

For peer review only

Table 2. Results of the abPG-SGA questionnaire completed 1 year after esophagectomy for cancer in relation to surgical technique in a Swedish population-based cohort.

AbPG-SGA	All	Open esophagectomy (OE)	Minimally invasive esophagectomy (MIE)	Hybrid minimally invasive esophagectomy (HMIE)
	Number (%)			
Number of patients	239	87	78	74
Body weight				
Reduced	25 (10)	11 (13)	7 (9)	7 (9)
Not changed	173 (72)	64 (74)	51 (65)	58 (78)
Increased	39 (16)	10 (11)	20 (26)	9 (12)
Food intake				
Unchanged	111 (46)	46 (53)	27 (35)	38 (51)
More than usual	8 (3)	5 (6)	1 (1)	2 (3)
Less than usual	116 (49)	34 (39)	49 (63)	33 (45)
If less than usual, I am now taking:				
Normal food but less amount	108 (45)	31 (36)	44 (56)	33 (45)
Little solid food	3 (1)	0 (0)	3 (4)	0 (0)
Only liquids/nutritional supplements	1 (0)	0 (0)	1 (1)	0 (0)
Very little of everything	1 (0)	1 (1)	0 (0)	0 (0)
Only tube feedings/nutritional by vein	3 (1)	2 (2)	1 (1)	0 (0)
Symptoms [1]				
No problem eating	107 (45)	35 (40)	31 (40)	41 (55)
No appetite	44 (18)	15 (17)	20 (26)	9 (12)
Nausea	34 (14)	15 (17)	14 (18)	5 (7)
Constipation	7 (3)	3 (3)	3 (4)	1 (1)
Mouth sores	3 (1)	0 (0)	1 (1)	2 (3)
Things tast funny or have no taste	19 (8)	6 (7)	9 (12)	4 (5)
Problems swallowing	35 (15)	16 (18)	13 (17)	6 (8)
Pain[2]	14 (6)	4 (5)	5 (6)	5 (7)
Other[3]	13 (5)	5 (6)	5 (6)	3 (4)
Vomiting	14 (6)	4 (5)	7 (9)	3 (4)
Diarrhea	20 (8)	8 (9)	8 (10)	4 (5)
Dry mouth	11 (5)	4 (5)	2 (3)	5 (7)
Smells bother me	8 (3)	2 (2)	5 (6)	1 (1)
Feel full quickly	77 (32)	25 (29)	29 (37)	23 (31)
Total number of symptoms reported:	299	107	121	71
Activities/function				

For peer review only

Normal with no limitations	143 (60)	50 (57)	43 (55)	50 (68)
Not my normal self	70 (29)	28 (32)	21 (27)	21 (28)
Not feeling up to most things	17 (7)	6 (7)	8 (10)	3 (4)
Able to be little active	6 (3)	1 (1)	5 (6)	0 (0)
Pretty much bed ridden	1 (0)	1 (1)	0 (0)	0 (0)
**Patients with malnutrition (total sum score)**	**71 (30)**	**22 (25)**	**33 (42)**	**16 (22)**

[1] Several answers possible. (Fatigue not included in the Swedish version of the abPG-SGA (17)).

[2] No patient gave information about where they had pain.

[3] For example, throat irritation, the food does not taste like before, feeling of strangulation in end of meal, tooth problems, dumping, concern because of family problems, swelling in throat.

For peer review only

Table 3. Associations between surgical technique for esophagectomy due to cancer and nutritional status and weight loss 1 year after surgery in a Swedish population-based cohort study.

	Open esophagectomy	Minimally invasive esophagectomy	Hybrid minimally invasive esophagectomy
	(Reference)	OR (95% CI)[1]	OR (95% CI)[1]
**Malnutrition – total score from abPG-SGA**	1.0	1.86 (0.91-3.82)	0.92 (0.42-2.04)
Reduced food intake	1.0	2.87 (1.47-5.61)	1.32 (0.68-2.59)
Symptoms reducing food intake[2]	1.0	1.32 (0.67-2.61)	0.93 (0.46-1.90)
Decreased activities/function	1.0	1.81 (0.65-5.02)	0.50 (0.12-2.11)
Weight			
Weight loss 6 months after surgery			
≤10%	1.0	1.26 (0.38-4.19)	1.14 (0.32-4.02)
>10%	1.0	1.51 (0.47-4.90)	1.59 (0.47-5.40)
Weight loss 1 year after surgery			
≤10%	1.0	3.08 (0.86-10.95)	0.90 (0.31-2.61)
>10%	1.0	2.06 (0.60-7.04)	0.96 (0.36-2.57)

[1] Adjusted for age, sex, Charlson's comorbidity index, preoperative BMI, pathological tumor stage, neoadjuvant therapy, enteral/parenteral nutrition support, and postoperative complications.

[2] Nausea, diarrhoea, dry mouth, problems swallowing, feel full quickly, fatigue, pain etc.

Abbreviations: OR, Odds ratio; CI, confidence interval.

1

For peer review only

FIGURES

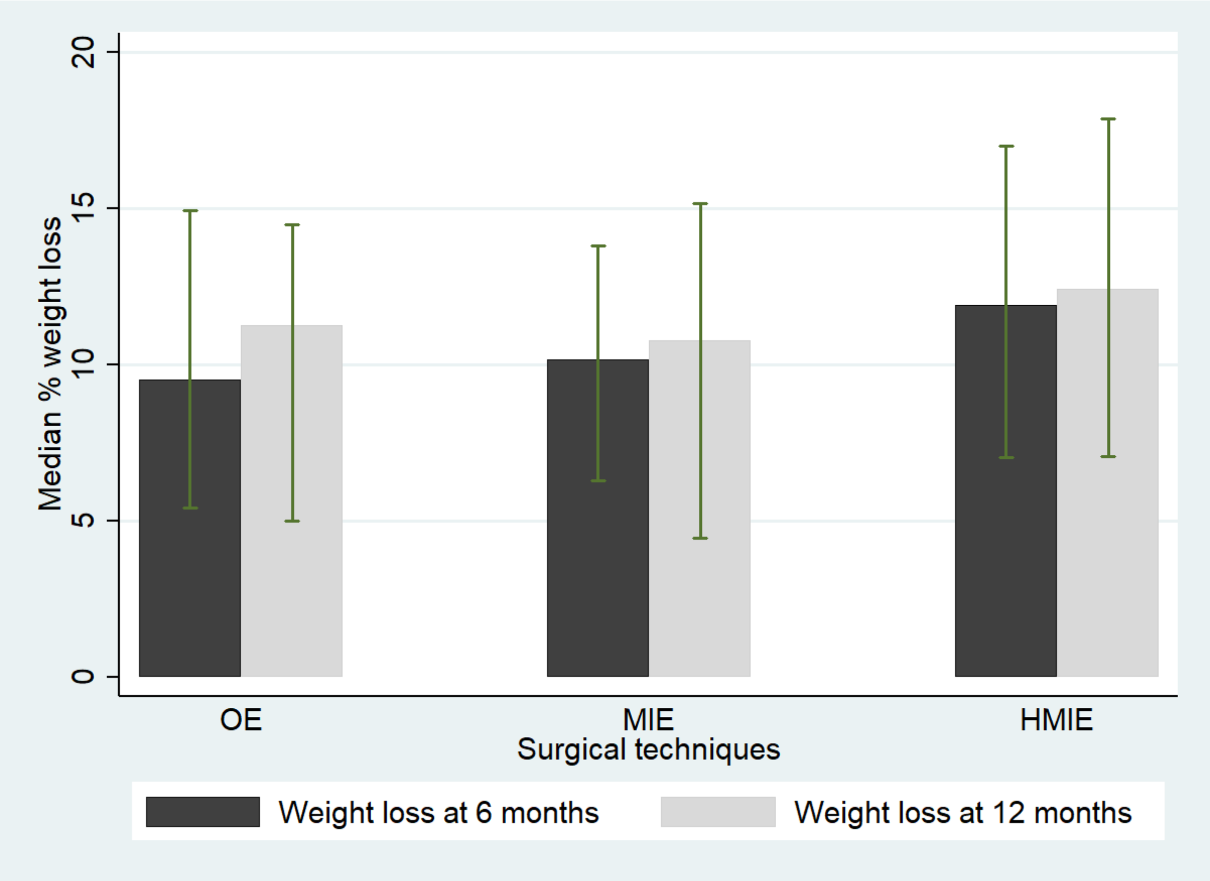

Figure 1.

Supplementary data to Minimally invasive surgical techniques for esophageal cancer and nutritional recovery – a prospective population-based cohort study.

Supplementary Table 1

Supplementary Table 2

Supplementary Figure 1

For peer review only

Supplementary Table 1. The proportion of minimally invasive esophagectomy, hybrid minimally invasive esophagectomy and open esophagectomy in the present study (OSCAR, Oesophageal Surgery on Cancer patients–Adaptation and Recovery), and in the population-based nationwide study of patients undergoing esophagectomy in Sweden (SESS, Swedish Esophageal Cancer Surgery Study) during the same time period.

	OSCAR-study	SESS-study
**Surgical approach**		
Minimally invasive	28 (21.7%)	76 (14.9%)
Hybrid minimally invasive	37 (28.6%)	133 (27.6%)
Open surgery	64 (49.6%)	273 (56.6%)

For peer review only

Supplementary Table 2. Explanatory analysis of anastomotic leak and intensive care unit and hospital stay on associations between surgical technique for esophagectomy due to cancer and nutritional status and weight loss 1 year after surgery in a Swedish population-based cohort study

	Open esophagectomy (Reference)	Minimally invasive esophagectomy OR (95% CI)[1]	Hybrid minimally invasive esophagectomy OR (95% CI)[1]
**Malnutrition – total score from abPG-SGA**	1.0	1.80 (0.84-3.86)	0.90 (0.39-2.07)
Reduced food intake	1.0	3.25 (1.60-6.57)	1.46 (0.73-2.93)
Symptoms reducing food intake[2]	1.0	1.35 (0.66-2.77)	0.96 (0.46-2.02)
Decreased activities/function	1.0	1.44 (0.48-4.35)	0.40 (0.09-1.77)
Weight			
Weight loss 6 months after surgery			
≤10%	1.0	1.14 (0.31-4.12)	1.04 (0.28-3.83)
>10%	1.0	1.49 (0.42-5.30)	1.56 (0.44-5.55)
Weight loss 1 year after surgery			
≤10%	1.0	2.86 (0.74-11.11)	0.73 (0.23-2.38)
>10%	1.0	1.56 (0.42-5.77)	0.84 (0.28-2.51)

[1] Adjusted for age, sex, Charlson's comorbidity index, preoperative BMI, pathological tumor stage, neoadjuvant therapy, enteral/parenteral nutrition support, postoperative complications, anastomotic leak, intensive care unit stay, and hospital stay.

[2] Nausea, diarrhoea, dry mouth, problems swallowing, feel full quickly, fatigue, pain etc.

Abbreviations: OR, Odds ratio; CI, confidence interval.

For peer review only

Supplementary Figure 1

Scored Patient-generated Subjective Global Assessment (PG-SGA)

History: Boxes 1 - 4 are designed to be completed by the patient.
[Boxes 1-4 are referred to as the PG-SGA Short Form (SF)]

Patient identification information

1. Weight *(see worksheet 1)*

In summary of my current and recent weight:

I currently weigh about _____ pounds
I am about _____ feet _____ inches tall

One month ago I weighed about _____ pounds
Six month ago I weighed about _____ pounds

During the past two weeks my weight has:

☐ Decreased (1) ☐ Not changed (0) ☐ Increased (0)

Box 1 ☐

2. Food intake: As compared to my normal intake, I would rate my food intake during the past month as

- ☐ Unchanged (0)
- ☐ More than usal (0)
- ☐ Less than usal (1)

I am now taking

- ☐ *Normal food* but less than normal amount (1)
- ☐ Little solid food (2)
- ☐ Only liquids (3)
- ☐ Only nutritional supplements (3)
- ☐ Very little of anything (4)
- ☐ Only tube feedings or only nutribution by vein (0)

Box 2 ☐

3. Symptoms: I have had the following problems that have kept me from eating enough during the past two weeks (check all that apply)

- ☐ No problem eating (0)
- ☐ No appetite, just did not feel like eating (3)
- ☐ Nausea (1)
- ☐ Constipation (1)
- ☐ Mouth sores (2)
- ☐ Things taste funny or have no taste (1)
- ☐ Problems swallowing (2)
- ☐ Pain; where? (3) ____________
- ☐ Other (1) ** ____________
- ☐ Vomiting (3)
- ☐ Diarrhea (3)
- ☐ Dry moth (1)
- ☐ Smells bother me (1)
- ☐ Feel full quickly (1)
- ☐ Fatigue (1)

Examples: Depression,money, or dental problems **Box 3 ☐

4. Activities and function:

Over the past month, I would generally rate my activity as:

- ☐ Normal with no limitations (0)
- ☐ Not my normal self, but able to be up and about with fairly normal activities (1)
- ☐ Not feeling up to most things, but in bed or chair less than half the day (2)
- ☐ Able to be little activity and spend most of the day in bed or chair (3)
- ☐ Pretty much bed ridden, rarely out of bed (3)

Box 4 ☐

The remainder of this form is to be completed by your doctor, nurse, dietitian, or therapist. Thank you.

Additive score of boxes 1-4 ☐ **A**

©FD Ottery 2005, 2006, 2015 v3.22.15
email: faithotterymdphd@aol.com or info@pt-global.org

only

STROBE Statement—Checklist of items that should be included in reports of ***cohort studies***

	Item No	Recommendation	Page No
**Title and abstract**	1	(*a*) Indicate the study's design with a commonly used term in the title or the abstract	1
		(*b*) Provide in the abstract an informative and balanced summary of what was done and what was found	2
**Introduction**			
Background/rationale	2	Explain the scientific background and rationale for the investigation being reported	4
Objectives	3	State specific objectives, including any prespecified hypotheses	4
**Methods**			
Study design	4	Present key elements of study design early in the paper	5
Setting	5	Describe the setting, locations, and relevant dates, including periods of recruitment, exposure, follow-up, and data collection	5
Participants	6	(*a*) Give the eligibility criteria, and the sources and methods of selection of participants. Describe methods of follow-up	5
		(*b*) For matched studies, give matching criteria and number of exposed and unexposed	
Variables	7	Clearly define all outcomes, exposures, predictors, potential confounders, and effect modifiers. Give diagnostic criteria, if applicable	5-7
Data sources/ measurement	8*	For each variable of interest, give sources of data and details of methods of assessment (measurement). Describe comparability of assessment methods if there is more than one group	5-7
Bias	9	Describe any efforts to address potential sources of bias	7-8,11
Study size	10	Explain how the study size was arrived at	9
Quantitative variables	11	Explain how quantitative variables were handled in the analyses. If applicable, describe which groupings were chosen and why	7-8
Statistical methods	12	(*a*) Describe all statistical methods, including those used to control for confounding	7-8
		(*b*) Describe any methods used to examine subgroups and interactions	N/A
		(*c*) Explain how missing data were addressed	9
		(*d*) If applicable, explain how loss to follow-up was addressed	
		(*e*) Describe any sensitivity analyses	
**Results**			
Participants	13*	(a) Report numbers of individuals at each stage of study—eg numbers potentially eligible, examined for eligibility, confirmed eligible, included in the study, completing follow-up, and analysed	9
		(b) Give reasons for non-participation at each stage	9
		(c) Consider use of a flow diagram	N/A
Descriptive data	14*	(a) Give characteristics of study participants (eg demographic, clinical, social) and information on exposures and potential confounders	9
		(b) Indicate number of participants with missing data for each variable of interest	N/A
		(c) Summarise follow-up time (eg, average and total amount)	N/A
Outcome data	15*	Report numbers of outcome events or summary measures over time	9-10

For peer review only - http://bmjopen.bmj.com/site/about/guidelines.xhtml

Main results	16	(*a*) Give unadjusted estimates and, if applicable, confounder-adjusted estimates and their precision (eg, 95% confidence interval). Make clear which confounders were adjusted for and why they were included	9-10
		(*b*) Report category boundaries when continuous variables were categorized	19
		(*c*) If relevant, consider translating estimates of relative risk into absolute risk for a meaningful time period	N/A
Other analyses	17	Report other analyses done—eg analyses of subgroups and interactions, and sensitivity analyses	10
**Discussion**			
Key results	18	Summarise key results with reference to study objectives	11
Limitations	19	Discuss limitations of the study, taking into account sources of potential bias or imprecision. Discuss both direction and magnitude of any potential bias	11
Interpretation	20	Give a cautious overall interpretation of results considering objectives, limitations, multiplicity of analyses, results from similar studies, and other relevant evidence	13
Generalisability	21	Discuss the generalisability (external validity) of the study results	11
**Other information**			
Funding	22	Give the source of funding and the role of the funders for the present study and, if applicable, for the original study on which the present article is based	14

*Give information separately for exposed and unexposed groups.

Note: An Explanation and Elaboration article discusses each checklist item and gives methodological background and published examples of transparent reporting. The STROBE checklist is best used in conjunction with this article (freely available on the Web sites of PLoS Medicine at http://www.plosmedicine.org/, Annals of Internal Medicine at http://www.annals.org/, and Epidemiology at http://www.epidem.com/). Information on the STROBE Initiative is available at http://www.strobe-statement.org.

For peer review only

For peer review only - http://bmjopen.bmj.com/site/about/guidelines.xhtml

BMJ Open

Minimally invasive surgical techniques for esophageal cancer and nutritional recovery – a prospective population-based cohort study.

Journal:	*BMJ Open*
Manuscript ID	bmjopen-2021-058763.R2
Article Type:	Original research
Date Submitted by the Author:	28-Jul-2022
Complete List of Authors:	Kauppila, Joonas; Karolinska University Hospital, Department of Molecular medicine and Surgery; University of Oulu, Department of Surgery<br>Rosenlund, Helen; Karolinska University Hospital, Department of Molecular medicine and Surgery; Danderyds Sjukhus AB, Department of Orthopaedics<br>Klevebro, Fredrik; Karolinska Institutet, Surgical Care Science<br>Johar, Asif; Karolinska Institutet, Department of Molecular medicine and Surgery<br>Anandavadivelan, Poorna; Karolinska Institutet, Department of Molecular medicine and Surgery<br>Mälberg, Kalle; Karolinska Institutet, Upper Gastrointestinal Research, Department of Molecular Medicine and Surgery<br>Lagergren, Pernilla; Karolinska Institute, Department of Molecular Medicine and Surgery
**Primary Subject Heading**:	Surgery
Secondary Subject Heading:	Nutrition and metabolism
Keywords:	Oesophageal disease < GASTROENTEROLOGY, SURGERY, NUTRITION & DIETETICS

SCHOLARONE™
Manuscripts

I, the Submitting Author has the right to grant and does grant on behalf of all authors of the Work (as defined in the below author licence), an exclusive licence and/or a non-exclusive licence for contributions from authors who are: i) UK Crown employees; ii) where BMJ has agreed a CC-BY licence shall apply, and/or iii) in accordance with the terms applicable for US Federal Government officers or employees acting as part of their official duties; on a worldwide, perpetual, irrevocable, royalty-free basis to BMJ Publishing Group Ltd ("BMJ") its licensees and where the relevant Journal is co-owned by BMJ to the co-owners of the Journal, to publish the Work in this journal and any other BMJ products and to exploit all rights, as set out in our licence.

The Submitting Author accepts and understands that any supply made under these terms is made by BMJ to the Submitting Author unless you are acting as an employee on behalf of your employer or a postgraduate student of an affiliated institution which is paying any applicable article publishing charge ("APC") for Open Access articles. Where the Submitting Author wishes to make the Work available on an Open Access basis (and intends to pay the relevant APC), the terms of reuse of such Open Access shall be governed by a Creative Commons licence – details of these licences and which Creative Commons licence will apply to this Work are set out in our licence referred to above.

Other than as permitted in any relevant BMJ Author's Self Archiving Policies, I confirm this Work has not been accepted for publication elsewhere, is not being considered for publication elsewhere and does not duplicate material already published. I confirm all authors consent to publication of this Work and authorise the granting of this licence.

For peer review only

Minimally invasive surgical techniques for esophageal cancer and nutritional recovery – a prospective population-based cohort study.

Joonas H. Kauppila PhD[1,2], Helen Rosenlund PhD[1,3], Fredrik Klevebro PhD[1], Asif Johar MSc[1], Poorna Anandavadivelan PhD[1], Kalle Mälberg MSc[1], Pernilla Lagergren PhD[1,4]

[1] Surgical Care Sciences, Department of Molecular medicine and Surgery, Karolinska Institutet, Karolinska University Hospital 171 76 Stockholm, Sweden.

[2] Department of Surgery, Oulu University Hospital, University of Oulu, Finland

[3] Clinical Nutrition and Dietetics, Department of Orthopaedics, Danderyd Hospital, Stockholm, Sweden.

[4] Department of Surgery & Cancer, Imperial College London, United Kingdom

Corresponding author and requests for reprints:

Joonas H. Kauppila, Surgical Care Science, Department of Molecular medicine and Surgery, Karolinska Institutet, Karolinska University Hospital, Retzius väg 13A, 171 77 Stockholm, Sweden; E-mail: joonas.kauppila@ki.se

Running title: Surgical techniques and nutritional recovery.

For peer review only

ABSTRACT (Word count 283)

Objectives: To explore whether the minimally invasive (MIE), or hybrid minimally invasive esophagectomy (HMIE) are associated with better nutritional status and less weight loss 1 year after surgery, compared with open esophagectomy (OE).

Design: Prospective cohort study

Setting: All patients undergoing esophagectomy for cancer in Sweden during 2013-2018.

Participants: A total of 424 patients alive at 1 year after surgery were eligible, and 281 completed the 1-year assessment. Of these, 239 had complete clinical data and were included in the analysis.

Primary and secondary outcome measure: The primary outcome was nutritional status at 1 year after surgery, assessed using the abPG-SGA questionnaire. The secondary outcomes included postoperative weight loss at 6 months and 1 year after surgery.

Results: Of the included patients, 78 underwent MIE, 74 HMIE while 87 patients underwent OE. The MIE group had the highest prevalence of malnutrition (42% vs. 22% after HMIE vs. 25% after OE), reduced food intake (63% vs 45% after HMIE vs. 39% after OE), symptoms reducing food intake (60% vs. 45% after HMIE vs. 60% after OE) and abnormal activities/function (45% vs. 32% after HMIE vs 43% after OE). After adjustment for confounders, MIE was associated with a statistically significant increased risk of reduced food intake 1 year after surgery (OR 2.87, 95% CI 1.47-5.61), compared to OE. Other outcomes were not statistically significantly different between the groups. No statistically significant associations were observed between surgical techniques and weight loss up to 1 year after surgery.

Conclusions: MIE was statistically significantly associated with reduced food intake 1 year after surgery. However, no differences were observed in weight loss between the surgical

For peer review only

techniques. Further studies on nutritional impact of surgical techniques in esophageal cancer are needed.

Keywords: esophageal cancer; minimally invasive surgery; nutrition

Strengths and limitations of this study:

- The strength of this study is the prospective and population-based study design with longitudinal collection of data according to a predefined study protocol.

- The relatively high inclusion rate and complete data are strengths.

- The abPG-SGA questionnaire is validated and commonly used to measure malnutrition in oncology patients.

- A weakness of this study was that all patients who died during 1 year after esophagectomy were not eligible.

For peer review only

INTRODUCTION

The mainstay of curative treatment for esophageal cancer is surgery which carries a high risk of postoperative complications and mortality (1). The surgery is often combined with preoperative neoadjuvant therapy (2). The traditional open esophagetomy (OE) with abdominal and thoracic incisions is being replaced by minimally invasive esophagectomy (MIE), performed using thoracoscopy and laparoscopy (3). MIE is associated with lower risk of postoperative complications, especially pulmonary, lower in-hospital mortality, shorter length of hospital stay, and lower 90-day mortality, compared to OE (4-7). MIE has also been associated with improved health-related quality of life (HRQL) (8, 9). Hybrid minimally invasive esophagectomy (HMIE) applies open surgery in combination with laparoscopic or thoracoscopic technique, e.g. a thoracotomy and laparoscopy. HMIE is associated less postoperative mortality and better HRQL, compared with OE (10).

Esophageal cancer is associated with significant nutritional problems and weight loss before and after treatment (11-13). Patients lose between 5-12% of their preoperative weight during the first 6 months after esophagectomy and a majority have lost more than 10% of their body weight 12 months postoperatively (14). However, the effect of MIE on nutritional recovery remains unclear. It was hypothesized that MIE could improve nutritional recovery after esophagectomy, compared to OE.

The aim of this study was to investigate the impact of surgical technique, used for esophagectomy for cancer, on nutritional status and weight loss up to 1 year after surgery in a population-based and nationwide setting.

For peer review only

MATERIALS AND METHODS

Study design

This project was based on the nationwide and prospective cohort entitled Oesophageal Surgery on Cancer patients–Adaptation and Recovery (OSCAR) (15). Patients who underwent surgery for esophageal cancer in Sweden from 1st January 2013 and onwards were included. Recruitment was done 1 year after surgery, and thus the inclusion started 1st January 2014. Eligible patients were identified from pathology units at eight treating hospitals in Sweden. An invitation letter was sent and within 1 week patients were contacted by telephone to get information regarding the project and to give their consent to participate. Arrangements for the first assessment were also made. A research nurse visited the patients in their homes for collection of patient-reported outcomes, objective measures of body weight and body composition. The OSCAR study was approved by the Regional Ethical Review Board in Stockholm, Sweden and all participants provided written informed consent.

view only

Clinical data at time of surgery was collected from medical records, which were reviewed according to a predefined study protocol to ensure uniformity: 1) Patient characteristics including age, sex, comorbidities, and weight at time of surgery; 2) Tumor histology, site/stage; 3) Treatment, including neoadjuvant or adjuvant oncological treatment, and surgical approach; 4) Postoperative complications classified according to the Clavien-Dindo scoring system (16).

Exposures

The following three types of surgery were used as exposures: 1) open transthoracic or transhiatal esophagectomy (OE; reference group), 2) minimally invasive esophagectomy (thoracoscopic-laparoscopic) (MIE), and 3) hybrid minimally invasive esophagectomy

(thoracoscopic/open abdomen or laparoscopic/open chest) (HMIE). Data on surgical technique was obtained from the medical records.

Outcomes

The primary outcome was malnutrition measured by the abPG-SGA (abbreviated patient-generated subjective global assessment) questionnaire (Supplementary Figure 1), that was completed by the patient at one time-point 1 year after surgery. This is a screening tool to detect patients at risk of malnutrition and is recommended for use in oncology patients (17, 18). The abPG-SGA is an abbreviated version of the PG-SGA questionnaire and has been found valid to detect malnutrition when used in an outpatient oncology clinic (19).

The abPG-SGA contains four sections which concern body weight, food intake, symptoms reducing food intake, and activities/function. The first section about body weight asks for the patient's current weight and height, weight at 1 and 6 months ago, and if the patient's weight has "decreased"/"increased"/"has been stable" during the last two weeks. The second section concerns food intake where the patient compares his/her food intake to their normal intake as "unchanged"/"more than usual"/"less than usual". The patient is then asked to relate his/her food intake to the habitual food intake prior to cancer symptoms/diagnosis (in the original questionnaire patients related food intake to 1 month ago). If the answer was "less than usual" the patient responded to the follow-up question: What type of food do you eat? with the following response alternatives "normal food but less than normal amount"/"little solid food"/"only liquids"/"only nutritional supplements"/"very little of anything"/"only tube feeding or parenteral nutrition". Section three covers symptoms that have kept the patient from eating enough during the past two weeks (several answers possible): "no problem"/"no appetite, just did not feel like eating"/"nausea"/"vomiting"/"constipation"/

For peer review only

“diarrhoea”/“mouth sores”/“dry mouth”/“things taste funny or have no taste”/“smells bother me”/“problems swallowing”/“feel full quickly”/“pain”/“other”. The forth section concerns activities/function and asks the patients to rate their general activity over the past months: “normal”/“not my normal self, but have fairly normal activities”/“not feeling up to things, in bed/chair half the day”/“able to be little active and spend most of the day in bed/chair”/“rarely out of bed”. The score from each part totals up a sum that determines the patient´s nutritional status. A higher score indicates more severe malnutrition (17, 19).

The secondary outcomes were postoperative weight loss between 1) time of surgery and 6 months postoperatively and 2) time of surgery and 1 year after surgery. The weight at surgery was collected from medical records, weight at 6 months was reported by the patient in the abPG-SGA questionnaire, and weight at 1 year was measured by the research nurse. Weight loss was calculated by comparing weight measures at surgery and weight at 6 months and at 1 year.

Statistical analysis

Logistic regression models were used to analyze the associations between surgical techniques (OE [reference group], MIE, HMIE) and malnutrition (yes, no) and weight loss at 6 months and at 1 year postoperatively. Weight loss was categorized as 1) No weight loss/weight gain, 2) ≤10% weight loss, 3) >10% weight loss. Results were presented as odds ratios (OR) with 95% confidence intervals (CI), adjusted for potential confounders: age (longitudinal), sex (male, female), Charlson’s comorbidity index (0, 1, ≥2), preoperative body mass index (BMI) (<25, ≥25), pathological tumor stage (0-I, II, III-IV), neoadjuvant therapy (yes, no), enteral/parenteral nutrition support during at least 1 week postoperatively (yes, no), and postoperative complications (Clavien-Dindo score [CDS]; low grade [0-II], high grade [III-

For peer review only

IV]). Furthermore, an explanatory analysis was conducted to further adjust for anastomotic leak defined as clinically significant or radiologically confirmed anastomotic leak (yes, no), intensive care unit stay (continuous) and hospital stay (continuous). Weight loss was also presented as median weight loss percentage, combined with the inter quartile range for each group. In the analyses of the abPG-SGA questionnaire, a summarized score of $\geq$6 was considered as malnutrition. For body weight, food intake, symptoms reducing food intake, and activities/function a score of $\geq$2 was considered as a lower/worse condition in each group, with a reference score of <2. A P-value <0.05 was considered statistically significant.

Patient and public involvement

The Surgical Care Science research partnership group including patients with esophageal cancer were involved in the planning of OSCAR cohort study. The partnership group patients were involved in discussions around the study questions, as well as on the potential outcome measures collected during the study. The partnership group were not involved in recruitment of participants, but have been involved in the dissemination of research findings and increasing public awareness of esophageal cancer. Regarding the present study, the research partnership group patients suggested nutritional as an important outcome requiring more studies. Findings from this study will be communicated to patients and healthcare professionals through conferences and patient meetings.

For peer review only

RESULTS

Participants

Between January 2013 and April 2018, 675 patients underwent esophageal cancer surgery in Sweden. Of these, 511 (76%) survived for at least 1 year, 85 were not reachable, and 2 were excluded because of cognitive impairment, leaving 424 patients eligible for inclusion. Of these, 281 (66%) patients completed the 1-year assessment and clinical data were available for 247 (58%) patients. In total, 239 patients were included in the analyses. A comparison of study population and a population-based nationwide study of patients undergoing esophagectomy in Sweden during the same period to examine the potential selection bias suggested no major difference in distribution of different surgical approaches in this study compared to population (Supplementary Table 1).

Patient characteristics

Patients were evenly distributed in the 3 surgical groups (OE 36%, MIE 33%, and HMIE 31%) (Table 1). The mean age was 66.3 (±8.7) years at time of surgery and most patients were male (87%). The distribution of preoperative BMI and weight loss were similar across the three groups. Preoperative neoadjuvant therapy was most often used in the HMIE group (84%), while a higher pathological tumor stage was more frequently seen in the MIE group (37%), compared to the other groups. Almost all patients had a gastric conduit, while few patients in the OE group had an esophagojejunostomy. A neck anastomosis was more common in the MIE group, compared to the OE and HMIE groups. Patients who underwent HMIE less often received enteral/parenteral nutrition for at least 1 week, compared to the other groups. Patients with jejunostomy received enteral feeding for 14 days in median.

For peer review only

Table 1. Distribution of patient characteristics in relation to the surgical technique used for esophagectomy for cancer in a Swedish population-based cohort study.

	All	Open esophagectomy (OE)	Minimally invasive esophagectomy (MIE)	Hybrid minimally invasive esophagectomy (HMIE)
	Number (%)			
Number of patients	239 (100)	87 (36)	78 (33)	74 (31)
Sex (male)	207 (87)	69 (79)	70 (90)	68 (92)
**At time of surgery**				
Age (mean + 1 SD)	66.3 + 8.7	65.8 + 8.4	66.2 + 9.8	66.9 + 7.9
Preoperative BMI (mean + 1 SD)				
<25	36 (15)	15 (17)	12 (15)	9 (12)
≥25	203 (63)	72 (83)	66 (85)	65 (88)
Preoperative weight loss				
No weight loss/weight gain	23 (10)	10 (11)	4 (5)	9 (12)
<10%	79 (33)	26 (30)	32 (41)	21 (28)
≥10%	137 (57)	51 (59)	42 (54)	44 (59)
Preoperative neoadjuvant treatment	188 (79)	68 (78)	58 (74)	62 (84)
Pathological tumor stage				
0-I	79 (33)	32 (37)	16 (21)	31 (42)
II	80 (33)	26 (30)	33 (42)	21 (28)
III-IV	80 (33)	29 (33)	29 (37)	22 (30)
Organ substitute				
Gastric tube	219 (92)	73 (84)	74 (95)	72 (97)
Esophagojejunostomy	10 (4)	8 (9)	1 (1)	1 (1)
Colonic interposition	2 (1)	1 (1)	1 (1)	0 (0)
Level of anastomosis				
Neck	38 (16)	9 (10)	20 (26)	9 (12)
Thorax	187 (78)	70 (80)	54 (69)	63 (85)
Abdomen	6 (3)	5 (6)	0 (0)	1 (1)
Charlson's comorbidity index				
0	116 (54)	43 (49)	37 (47)	36 (49)
1	77 (28)	27 (31)	23 (29)	27 (36)
≥2	46 (18)	17 (20)	18 (23)	11 (15)
Enteral/parenteral nutrition	175 (73)	68 (78)	60 (77)	47 (64)
Postoperative complications				
Low grade (CDS 0-II)	144 (60)	51 (59)	44 (56)	49 (66)
High grade (CDS III-IV)	95 (40)	36 (41)	34 (44)	25 (34)
Anastomotic leak	41 (17)	9 (10)	21 (27)	11 (15)
ICU stay (median, [IQR])	2 [1-5]	3 [1-6]	3 [1-6]	2 [1-3]
Hospital stay (median, [IQR])	16 [12-25]	16 [13-26]	16.5 [12-29]	15 [11-20]

SD=Standard Deviation, BMI=Body Mass Index, CDS=Clavien-Dindo Score.

For peer review only

Nutritional status

One third (30%) of all patients had malnutrition 1 year postoperatively, based on the abPG-SGA (Table 2), and it was highest in the MIE group (42%), compared to the OE (25%) and the HMIE (22%) groups. The MIE group had the highest prevalence of reduced food intake (63% vs 45% after HMIE vs. 39% after OE), symptoms reducing food intake (60% vs. 45% after HMIE vs. 60% after OE). The number of reported symptoms reducing food intake (each symptom counted once for each patient, one patient could have multiple symptoms) in the MIE group was higher in the MIE group (121), compared to HMIE (71) and OE (107). Furthermore, abnormal activities/function were more common after MIE (45% vs. 32% after HMIE vs 43% after OE). In multivariable analysis, the point estimate for the risk of malnutrition was high after MIE, compared to OE (OR_{adj} 1.86, 95% CI 0.91-3.82), but this difference was statistically non-significant. A statistically significant association was observed between MIE and reduced food intake at 1 year after surgery (OR_{adj} 2.87, 95% CI 1.47-5.61), compared to OE (Table 3). No associations were observed between surgical technique and symptoms reducing food intake or activities/function.

For peer review only

Table 2. Results of the abPG-SGA questionnaire completed 1 year after esophagectomy for cancer in relation to surgical technique in a Swedish population-based cohort.

AbPG-SGA	All	Open esophagectomy (OE)	Minimally invasive esophagectomy (MIE)	Hybrid minimally invasive esophagectomy (HMIE)
	Number (%)			
Number of patients	239	87	78	74
Body weight				
Reduced	25 (10)	11 (13)	7 (9)	7 (9)
Not changed	173 (72)	64 (74)	51 (65)	58 (78)
Increased	39 (16)	10 (11)	20 (26)	9 (12)
Food intake				
Unchanged	111 (46)	46 (53)	27 (35)	38 (51)
More than usual	8 (3)	5 (6)	1 (1)	2 (3)
Less than usual	116 (49)	34 (39)	49 (63)	33 (45)
If less than usual, I am now taking:				
Normal food but less amount	108 (45)	31 (36)	44 (56)	33 (45)
Little solid food	3 (1)	0 (0)	3 (4)	0 (0)
Only liquids/nutritional supplements	1 (0)	0 (0)	1 (1)	0 (0)
Very little of everything	1 (0)	1 (1)	0 (0)	0 (0)
Only tube feedings/nutritional by vein	3 (1)	2 (2)	1 (1)	0 (0)
Symptoms [1]				
No problem eating	107 (45)	35 (40)	31 (40)	41 (55)
No appetite	44 (18)	15 (17)	20 (26)	9 (12)
Nausea	34 (14)	15 (17)	14 (18)	5 (7)
Constipation	7 (3)	3 (3)	3 (4)	1 (1)
Mouth sores	3 (1)	0 (0)	1 (1)	2 (3)
Things tast funny or have no taste	19 (8)	6 (7)	9 (12)	4 (5)
Problems swallowing	35 (15)	16 (18)	13 (17)	6 (8)
Pain[2]	14 (6)	4 (5)	5 (6)	5 (7)
Other[3]	13 (5)	5 (6)	5 (6)	3 (4)
Vomiting	14 (6)	4 (5)	7 (9)	3 (4)
Diarrhea	20 (8)	8 (9)	8 (10)	4 (5)
Dry mouth	11 (5)	4 (5)	2 (3)	5 (7)
Smells bother me	8 (3)	2 (2)	5 (6)	1 (1)
Feel full quickly	77 (32)	25 (29)	29 (37)	23 (31)
Total number of symptoms reported:	299	107	121	71
Activities/function				
Normal with no limitations	143 (60)	50 (57)	43 (55)	50 (68)

For peer review only

Not my normal self	70 (29)	28 (32)	21 (27)	21 (28)
Not feeling up to most things	17 (7)	6 (7)	8 (10)	3 (4)
Able to be little active	6 (3)	1 (1)	5 (6)	0 (0)
Pretty much bed ridden	1 (0)	1 (1)	0 (0)	0 (0)
**Patients with malnutrition (total sum score)**	**71 (30)**	**22 (25)**	**33 (42)**	**16 (22)**

[1] Several answers possible. (Fatigue not included in the Swedish version of the abPG-SGA (17)).

[2] No patient gave information about where they had pain.

[3] For example, throat irritation, the food does not taste like before, feeling of strangulation in end of meal, tooth problems, dumping, concern because of family problems, swelling in throat.

For peer review only

Table 3. Associations between surgical technique for esophagectomy due to cancer and nutritional status and weight loss 1 year after surgery in a Swedish population-based cohort study.

	Open esophagectomy	Minimally invasive esophagectomy	Hybrid minimally invasive esophagectomy
	(Reference)	OR (95% CI)[1]	OR (95% CI)[1]
**Malnutrition – total score from abPG-SGA**	1.0	1.86 (0.91-3.82)	0.92 (0.42-2.04)
Reduced food intake	1.0	2.87 (1.47-5.61)	1.32 (0.68-2.59)
Symptoms reducing food intake[2]	1.0	1.32 (0.67-2.61)	0.93 (0.46-1.90)
Decreased activities/function	1.0	1.81 (0.65-5.02)	0.50 (0.12-2.11)
Weight			
Weight loss 6 months after surgery			
≤10%	1.0	1.26 (0.38-4.19)	1.14 (0.32-4.02)
>10%	1.0	1.51 (0.47-4.90)	1.59 (0.47-5.40)
Weight loss 1 year after surgery			
≤10%	1.0	3.08 (0.86-10.95)	0.90 (0.31-2.61)
>10%	1.0	2.06 (0.60-7.04)	0.96 (0.36-2.57)

[1] Adjusted for age, sex, Charlson's comorbidity index, preoperative BMI, pathological tumor stage, neoadjuvant therapy, enteral/parenteral nutrition support, and postoperative complications.

[2] Nausea, diarrhoea, dry mouth, problems swallowing, feel full quickly, fatigue, pain etc.

Abbreviations: OR, Odds ratio; CI, confidence interval.

For peer review only

In a sensitivity analysis the level of anastomosis was included in the model, but the results remained the same, except that the point estimate of reduced food intake after MIE was increased to 3.13 (95% CI 1.53-6.39). Furthermore, in an attempt to minimize the risk that patients in the OE group reconstructed with jejunal conduit affected the outcomes, these 8 patients were excluded from the analyses, but the results remain unchanged. Lastly, adjustment for anastomotic leak, intensive care unit stay and hospital stay in the explanatory analysis did not change the point estimates compared to the main analysis (Supplementary Table 2).

Weight loss

The patients lost most weight during the first 6 months postoperatively in all groups and it was most pronounced after HMIE, compared to both OE and MIE. A similar pattern was observed for weight loss at 1 year after surgery (Figure 1). Surgical technique was not associated with weight loss at 6 months or 1 year after surgery (Table 3). Additional adjustment for anastomotic leak, intensive care unit stay and hospital stay did not change the estimates (Supplementary Table 2).

For peer review only

DISCUSSION

The results of this population-based nationwide cohort study suggest that patients operated with MIE and HMIE had no increased risk in malnutrition compared to OE, but those operated with MIE were more likely to have reduced food intake 1 year postoperatively, compared to OE. No association between surgical technique and weight loss up to 1 year postoperatively was present.

The strength of this study is the prospective and population-based study design with longitudinal collection of data. Clinical data at time of surgery was collected from medical records and reviewed according to a predefined study protocol to ensure consistency and uniformity of the data. The cohort had relatively high inclusion rate, and completeness of the abPG-SGA questionnaire in survivors 1 year postoperatively was very high. The abPG-SGA questionnaire is a validated and commonly-used instrument that has been reported to be a valid tool to measure malnutrition in oncology patients (19). The results may be generalizable to hospitals in Western countries where these surgical techniques are used. Missing data is in general low when collected by a research nurse in patients' homes. Weaknessess of this study include that all patients who died during 1 year after esophagectomy were not eligible for recruitment in the study, and that there is potential selection bias at the inclusion time of 1 year after surgery despite the distributions of surgical approaches being similar in this study and the population. Moreover, the patients´ self-reported weight measure could be biased, although this is assumed to be a minor problem since keeping track of weight is a central concern among these patients. In the section about food intake in the abPG-SGA, the nurse asked these patients to relate their actual food intake to their habitual eating prior to diagnosis, instead of 1 month ago, to make it more suitable for this patient group. This may give a more

For peer review only

correct picture of the nutritional status in this group since comparing to 1 month ago would not have shown any difference because their eating problems started at time of surgery or earlier.

Previous studies comparing malnutrition between MIE and OE are sparse, as very few have looked at malnutrition in the long term, and none using the abPG-SGA questionnaire. The previous studies assessing malnutrition have used weight loss or BMI as a proxy for nutritional status(20, 21) or laboratory values (6, 22). In an Italian case-control study, serum albumin levels were higher at day 1, 3, and 8 in patients undergoing HMIE, compared to OE.(22) An American study suggested earlier oral intake in patients who underwent MIE, compared to OE(6). The results from the current study suggest that MIE has a greater negative effect on the patients´ food intake, compared to OE, which is the opposite of what we hypothesized *a priori*. However, there was no significant difference in malnutrition, or other nutritional symptoms between the surgical techniques. Based on previous research, MIE should lead to less postoperative complications and shorter length of hospital stay, compared to OE (5-7). In the present study, major complications were similar between the groups and likely not a relevant factor in food intake symptoms. Patients undergoing MIE had higher occurrence of anastomotic leaks (27%) than those operated with OE (10%) or HMIE (15%). The higher percentages of anastomotic leaks are most likely related to the ongoing learning curve of MIE and HMIE in Sweden during the study period. A previous dutch study suggested that there are more anastomotic leaks (19%) during the learning phase of MIE compared to after completed learning (5%).(23) Therefore, it could be speculated that anastomotic leaks related to MIE and HMIE would explain the observed differences in food intake. However, an additional adjustment for anastomotic leak, intensive care unit stay, and hospital stay in the explanatory analysis did not result in major changes in the point estimates

For peer review only

compared to the main analysis. Unfortunately no data on the management of anastomotic leaks was available in this study, and the possible effects of sequelae of anastomotic leaks could not be furhter explored. On the other hand, neck anastomosis is known to be associated with more anastomotic strictures, compared to chest anastomosis, which might affect the food intake(24). As the MIE group had more neck anastomoses than HMIE and OE groups, a sensitivity analysis adjusted for anastomotic location was conducted but the results were no different from the main analysis. The conduit used for reconstruction might also affect food intake, but excluding jejunal interposition grafts did not change the results. Anastomotic techniques (i.e., handsewn vs circular stapled vs. linear stapled) might also differ between MIE, HMIE and OE. For example, end-to-side stapled and hand-sewn, as well as linear stapled side-to-side anastomoses have been described in Sweden(25, 26). However, these differences remain speculative, as this data was not available, and further studies are needed. Lastly, early oral feeding is central to any Enhanced Recovery After Surgery (ERAS) programs, (27) and could impact the long-term nutritional recovery of these patients. However, participation in ERAS programs or the nutritional protocols in the early postoperative period were not available in the dataset. On the other hand, early oral nutrition was not associated to weight loss compared to jejunostomy after 1 month past surgery in a Dutch cohort study of 114 MIE patients. (28) As esophageal cancer strongly affects the patients´ food intake before and after surgery, the associated weight loss is often substantial(29). Some previous studies have investigated how surgical technique affects patients´ weight in the long-term. A Swedish clinical study observed no difference in weight loss during the first postoperative year in patients who underwent MIE, compared to OE (20). Our results suggested a higher weight loss in the HMIE group, both at 6 months and 1 year postoperatively, but this difference was non-significant in the adjusted analyses. In addition to no nutritional differences between the surgical methods, the results could be explained by too

For peer review only

small number of patients in the analysis. However, the weight loss in the OE group was increasing up to 1 year after surgery, and studies with longer-term follow-up might be warranted.

Even though there were no major differences in malnutrition between minimally invasive and open esophagectomy, a striking finding of this study is that one third (30%) of all included patients were malnourished at 1 year after surgery. This highlights the importance of nutritional support and long-term follow-up from the health care for patients who undergo esophagectomy. Since esophagectomy results in a permanent anatomical change of the upper gastrointestinal tract, besides nutritional strategies in the immediate postoperative phase such as early enteral feeding, nutritional interventions for postoperative complications, attention to long-term nutritional intake and status are also of high importance(29). While surgical technique might not be of high importance, the impact of anastomotic technique on nutrition in esophageal cancer patients should be further evaluated. With improved knowledge, the utility of long-term dietitian support in different patient groups could be clarified.

In conclusion, this population-based prospective cohort study suggests that while there was no increased risk of malnutrition between minimally invasive compared to open esophagectomy, MIE is associated to reduced food intake 1 year postoperatively. No significant differences were present between surgical technique and weight loss. Further studies on nutritional impact of surgical techniques in esophageal cancer are needed.

ACKNOWLEDGEMENTS

For peer review only

We thank all study participants and the members of the Surgical Care Science patient research partnership group for their contribution to the OSCAR study.

Contributorship statement

JHK, HR, FK, PA, and PL contributed with input in designing the study. Data collection and assembly of data were performed by KM and PL. Data analysis was conducted by AJ. All authors (JHK, HR, FK, AJ, PA, KM, and PL) contributed in interpreting the results, manuscript writing and approval of the final version of the manuscript.

Competing interests

The authors state no conflicts of interest.

Funding statement

We also would like to thank the Cancerfonden (grant number 180685), the Cancer Research Funds of Radiumhemmet (grant number 171103), and the Stockholm County Council (grant number LS 2018–1157) for financial support. Pernilla Lagergren is supported by the NIHR Imperial Biomedical Research Centre (BRC) for her position at Imperial College London, London, United Kingdom. The funding sources had no role in the design and conduct of the data collection, management, analysis and interpretation of the data or preparation review or approval of the manuscript.

Data Sharing Statement

All data underlying the findings are presented in the manuscript. The datasets generated and/or analyzed in the current study will not be publicly available due to the ethical review act but will be available from the principal investigators of the study on reasonable request.

For peer review only

Ethics Approval Statement

The OSCAR study was approved by the Regional Ethical Review Board in Stockholm, Sweden (2013/844-31/1) and all participants provided written informed consent.

For peer review only

REFERENCES

1. Markar SR, Karthikesalingam A, Low DE. Outcomes assessment of the surgical management of esophageal cancer in younger and older patients. Ann Thorac Surg. 2012;94(5):1652-8.
2. Lagergren J, Smyth E, Cunningham D, Lagergren P. Oesophageal cancer. Lancet. 2017;390(10110):2383-96.
3. Lazzarino AI, Nagpal K, Bottle A, Faiz O, Moorthy K, Aylin P. Open versus minimally invasive esophagectomy: trends of utilization and associated outcomes in England. Ann Surg. 2010;252(2):292-8.
4. Briez N, Piessen G, Torres F, Lebuffe G, Triboulet JP, Mariette C. Effects of hybrid minimally invasive oesophagectomy on major postoperative pulmonary complications. Br J Surg. 2012;99(11):1547-53.
5. Yibulayin W, Abulizi S, Lv H, Sun W. Minimally invasive oesophagectomy versus open esophagectomy for resectable esophageal cancer: a meta-analysis. World J Surg Oncol. 2016;14(1):304.
6. Tapias LF, Mathisen DJ, Wright CD, Wain JC, Gaissert HA, Muniappan A, et al. Outcomes With Open and Minimally Invasive Ivor Lewis Esophagectomy After Neoadjuvant Therapy. Ann Thorac Surg. 2016;101(3):1097-103.
7. Kauppila JH, Helminen O, Kyto V, Gunn J, Lagergren J, Sihvo E. Short-Term Outcomes Following Minimally Invasive and Open Esophagectomy: A Population-Based Study from Finland and Sweden. Ann Surg Oncol. 2018;25(1):326-32.
8. Kauppila JH, Xie S, Johar A, Markar SR, Lagergren P. Meta-analysis of health-related quality of life after minimally invasive versus open oesophagectomy for oesophageal cancer. Br J Surg. 2017;104(9):1131-40.
9. Maas KW, Cuesta MA, van Berge Henegouwen MI, Roig J, Bonavina L, Rosman C, et al. Quality of Life and Late Complications After Minimally Invasive Compared to Open Esophagectomy: Results of a Randomized Trial. World J Surg. 2015;39(8):1986-93.
10. Mariette C, Markar SR, Dabakuyo-Yonli TS, Meunier B, Pezet D, Collet D, et al. Hybrid Minimally Invasive Esophagectomy for Esophageal Cancer. N Engl J Med. 2019;380(2):152-62.
11. Haverkort EB, Binnekade JM, Busch OR, van Berge Henegouwen MI, de Haan RJ, Gouma DJ. Presence and persistence of nutrition-related symptoms during the first year following esophagectomy with gastric tube reconstruction in clinically disease-free patients. World J Surg. 2010;34(12):2844-52.
12. Anandavadivelan P, Lagergren P. Cachexia in patients with oesophageal cancer. Nat Rev Clin Oncol. 2016;13(3):185-98.
13. Bozzetti F. Nutritional support in patients with oesophageal cancer. Support Care Cancer. 2010;18 Suppl 2:S41-50.
14. Baker M, Halliday V, Williams RN, Bowrey DJ. A systematic review of the nutritional consequences of esophagectomy. Clin Nutr. 2016;35(5):987-94.
15. Schandl A, Johar A, Anandavadivelan P, Vikstrom K, Malberg K, Lagergren P. Patient-reported outcomes 1 year after oesophageal cancer surgery. Acta Oncol. 2020:1-7.
16. Clavien PA, Barkun J, de Oliveira ML, Vauthey JN, Dindo D, Schulick RD, et al. The Clavien-Dindo classification of surgical complications: five-year experience. Ann Surg. 2009;250(2):187-96.

For peer review only

17. Persson C, Sjoden PO, Glimelius B. The Swedish version of the patient-generated subjective global assessment of nutritional status: gastrointestinal vs urological cancers. Clin Nutr. 1999;18(2):71-7.
18. Bauer J, Capra S, Ferguson M. Use of the scored Patient-Generated Subjective Global Assessment (PG-SGA) as a nutrition assessment tool in patients with cancer. Eur J Clin Nutr. 2002;56(8):779-85.
19. Gabrielson DK, Scaffidi D, Leung E, Stoyanoff L, Robinson J, Nisenbaum R, et al. Use of an abridged scored Patient-Generated Subjective Global Assessment (abPG-SGA) as a nutritional screening tool for cancer patients in an outpatient setting. Nutr Cancer. 2013;65(2):234-9.
20. Ericson J, Lundell L, Klevebro F, Kamiya S, Nilsson M, Rouvelas I. Long-term weight development after esophagectomy for cancer-comparison between open Ivor-Lewis and minimally invasive surgical approaches. Dis Esophagus. 2018.
21. Hynes O, Anandavadivelan P, Gossage J, Johar AM, Lagergren J, Lagergren P. The impact of pre- and post-operative weight loss and body mass index on prognosis in patients with oesophageal cancer. Eur J Surg Oncol. 2017;43(8):1559-65.
22. Scarpa M, Cavallin F, Saadeh LM, Pinto E, Alfieri R, Cagol M, et al. Hybrid minimally invasive esophagectomy for cancer: impact on postoperative inflammatory and nutritional status. Dis Esophagus. 2016;29(8):1064-70.
23. van Workum F, Stenstra M, Berkelmans GHK, Slaman AE, van Berge Henegouwen MI, Gisbertz SS, et al. Learning Curve and Associated Morbidity of Minimally Invasive Esophagectomy: A Retrospective Multicenter Study. Ann Surg. 2019;269(1):88-94.
24. van Workum F, van der Maas J, van den Wildenberg FJ, Polat F, Kouwenhoven EA, van Det MJ, et al. Improved Functional Results After Minimally Invasive Esophagectomy: Intrathoracic Versus Cervical Anastomosis. Ann Thorac Surg. 2017;103(1):267-73.
25. Rutegard M, Lagergren J, Rouvelas I, Lindblad M, Blazeby JM, Lagergren P. Population-based study of surgical factors in relation to health-related quality of life after oesophageal cancer resection. Br J Surg. 2008;95(5):592-601.
26. Nilsson M, Kamiya S, Lindblad M, Rouvelas I. Implementation of minimally invasive esophagectomy in a tertiary referral center for esophageal cancer. J Thorac Dis. 2017;9(Suppl 8):S817-S25.
27. Weimann A, Braga M, Carli F, Higashiguchi T, Hubner M, Klek S, et al. ESPEN guideline: Clinical nutrition in surgery. Clin Nutr. 2017;36(3):623-50.
28. Berkelmans GHK, Fransen L, Weijs TJ, Lubbers M, Nieuwenhuijzen GAP, Ruurda JP, et al. The long-term effects of early oral feeding following minimal invasive esophagectomy. Dis Esophagus. 2018;31(1):1-8.
29. Steenhagen E, van Vulpen JK, van Hillegersberg R, May AM, Siersema PD. Nutrition in peri-operative esophageal cancer management. Expert Rev Gastroenterol Hepatol. 2017;11(7):663-72.

For peer review only

FIGURE LEGENDS

Figure 1. Median (%) weight loss at 6 months and at 1 year after surgery in patients who underwent esophagectomy with different surgical techniques; OE (open esophagectomy), MIE (minimally invasive esophagectomy), HMIE (hybrid minimally invasive esophagectomy)

For peer review only

FIGURES

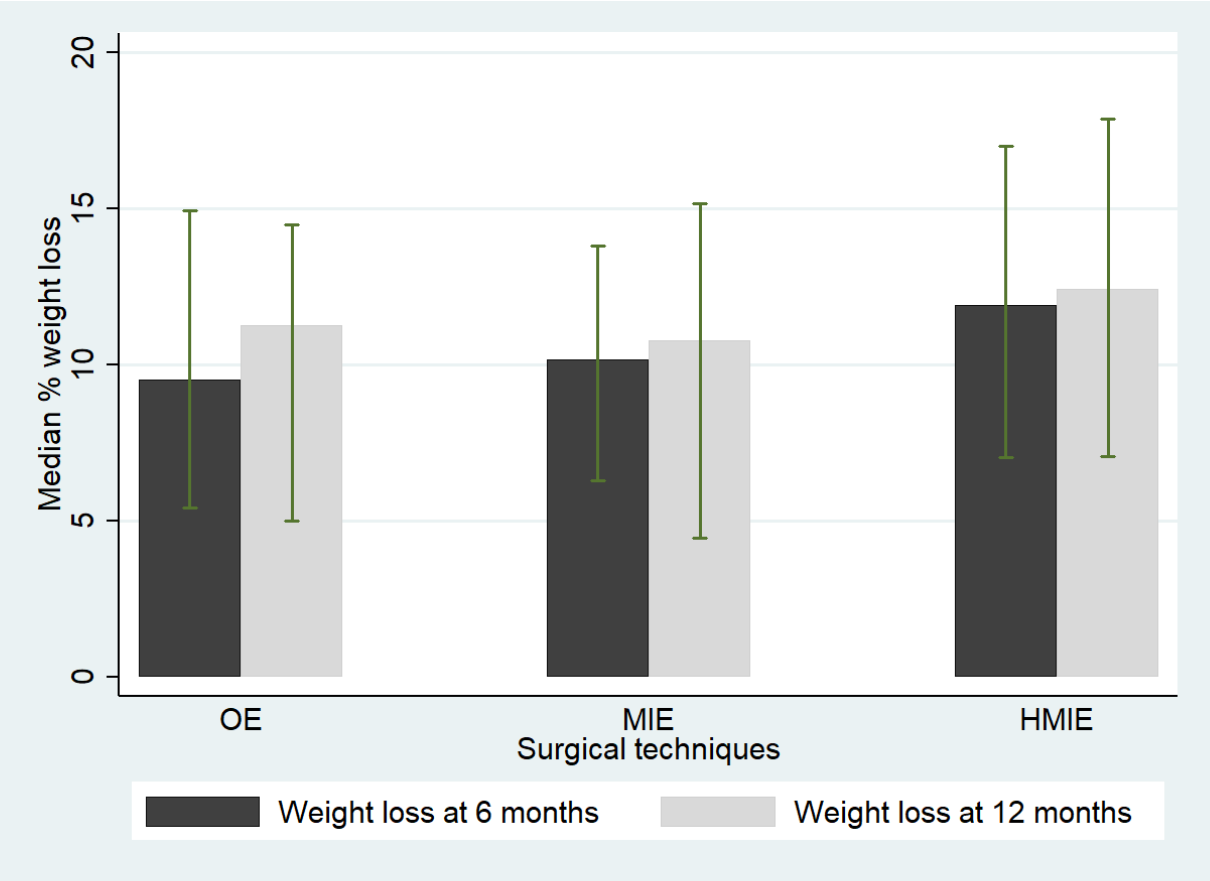

Figure 1.

Supplementary data to Minimally invasive surgical techniques for esophageal cancer and nutritional recovery – a prospective population-based cohort study.

Supplementary Table 1

Supplementary Table 2

Supplementary Figure 1

For peer review only

Supplementary Table 1. The proportion of minimally invasive esophagectomy, hybrid minimally invasive esophagectomy and open esophagectomy in the present study (OSCAR, Oesophageal Surgery on Cancer patients–Adaptation and Recovery), and in the population-based nationwide study of patients undergoing esophagectomy in Sweden (SESS, Swedish Esophageal Cancer Surgery Study) during the same time period.

	OSCAR-study	SESS-study
**Surgical approach**		
Minimally invasive	28 (21.7%)	76 (14.9%)
Hybrid minimally invasive	37 (28.6%)	133 (27.6%)
Open surgery	64 (49.6%)	273 (56.6%)

For peer review only

Supplementary Table 2. Explanatory analysis of anastomotic leak and intensive care unit and hospital stay on associations between surgical technique for esophagectomy due to cancer and nutritional status and weight loss 1 year after surgery in a Swedish population-based cohort study

	Open esophagectomy	Minimally invasive esophagectomy	Hybrid minimally invasive esophagectomy
	(Reference)	OR (95% CI)[1]	OR (95% CI)[1]
**Malnutrition – total score from abPG-SGA**	1.0	1.80 (0.84-3.86)	0.90 (0.39-2.07)
Reduced food intake	1.0	3.25 (1.60-6.57)	1.46 (0.73-2.93)
Symptoms reducing food intake[2]	1.0	1.35 (0.66-2.77)	0.96 (0.46-2.02)
Decreased activities/function	1.0	1.44 (0.48-4.35)	0.40 (0.09-1.77)
Weight			
Weight loss 6 months after surgery			
≤10%	1.0	1.14 (0.31-4.12)	1.04 (0.28-3.83)
>10%	1.0	1.49 (0.42-5.30)	1.56 (0.44-5.55)
Weight loss 1 year after surgery			
≤10%	1.0	2.86 (0.74-11.11)	0.73 (0.23-2.38)
>10%	1.0	1.56 (0.42-5.77)	0.84 (0.28-2.51)

[1] Adjusted for age, sex, Charlson's comorbidity index, preoperative BMI, pathological tumor stage, neoadjuvant therapy, enteral/parenteral nutrition support, postoperative complications, anastomotic leak, intensive care unit stay, and hospital stay.

[2] Nausea, diarrhoea, dry mouth, problems swallowing, feel full quickly, fatigue, pain etc.

Abbreviations: OR, Odds ratio; CI, confidence interval.

For peer review only

Supplementary Figure 1

Scored Patient-generated Subjective Global Assessment (PG-SGA)

History: Boxes 1 - 4 are designed to be completed by the patient.
[Boxes 1-4 are referred to as the PG-SGA Short Form (SF)]

Patient identification information

1. Weight *(see worksheet 1)*

In summary of my current and recent weight:

I currently weigh about _____ pounds
I am about _____ feet _____ inches tall

One month ago I weighed about _____ pounds
Six month ago I weighed about _____ pounds

During the past two weeks my weight has:
☐ Decreased (1) ☐ Not changed (0) ☐ Increased (0)

Box 1 ☐

2. Food intake: As compared to my normal intake, I would rate my food intake during the past month as
- ☐ Unchanged (0)
- ☐ More than usal (0)
- ☐ Less than usal (1)

I am now taking
- ☐ *Normal food* but less than normal amount (1)
- ☐ Little solid food (2)
- ☐ Only liquids (3)
- ☐ Only nutritional supplements (3)
- ☐ Very little of anything (4)
- ☐ Only tube feedings or only nutribution by vein (0)

Box 2 ☐

3. Symptoms: I have had the following problems that have kept me from eating enough during the past two weeks (check all that apply)
- ☐ No problem eating (0)
- ☐ No appetite, just did not feel like eating (3)
- ☐ Vomiting (3)
- ☐ Nausea (1)
- ☐ Diarrhea (3)
- ☐ Constipation (1)
- ☐ Dry moth (1)
- ☐ Mouth sores (2)
- ☐ Smells bother me (1)
- ☐ Things taste funny or have no taste (1)
- ☐ Feel full quickly (1)
- ☐ Problems swallowing (2)
- ☐ Fatigue (1)
- ☐ Pain; where? (3) ____________________
- ☐ Other (1) ** ____________________

Examples: Depression,money, or dental problems **Box 3 ☐

4. Activities and function:
Over the past month, I would generally rate my activity as:
- ☐ Normal with no limitations (0)
- ☐ Not my normal self, but able to be up and about with fairly normal activities (1)
- ☐ Not feeling up to most things, but in bed or chair less than half the day (2)
- ☐ Able to be little activity and spend most of the day in bed or chair (3)
- ☐ Pretty much bed ridden, rarely out of bed (3)

Box 4 ☐

The remainder of this form is to be completed by your doctor, nurse, dietitian, or therapist. Thank you.

Additive score of boxes 1-4 ☐ **A**

©FD Ottery 2005, 2006, 2015 v3.22.15
email: faithotterymdphd@aol.com or info@pt-global.org

only

STROBE Statement—Checklist of items that should be included in reports of ***cohort studies***

	Item No	Recommendation	Page No
**Title and abstract**	1	(*a*) Indicate the study's design with a commonly used term in the title or the abstract	1
		(*b*) Provide in the abstract an informative and balanced summary of what was done and what was found	2
**Introduction**			
Background/rationale	2	Explain the scientific background and rationale for the investigation being reported	4
Objectives	3	State specific objectives, including any prespecified hypotheses	4
**Methods**			
Study design	4	Present key elements of study design early in the paper	5
Setting	5	Describe the setting, locations, and relevant dates, including periods of recruitment, exposure, follow-up, and data collection	5
Participants	6	(*a*) Give the eligibility criteria, and the sources and methods of selection of participants. Describe methods of follow-up	5
		(*b*) For matched studies, give matching criteria and number of exposed and unexposed	
Variables	7	Clearly define all outcomes, exposures, predictors, potential confounders, and effect modifiers. Give diagnostic criteria, if applicable	5-7
Data sources/ measurement	8*	For each variable of interest, give sources of data and details of methods of assessment (measurement). Describe comparability of assessment methods if there is more than one group	5-7
Bias	9	Describe any efforts to address potential sources of bias	7-8,11
Study size	10	Explain how the study size was arrived at	9
Quantitative variables	11	Explain how quantitative variables were handled in the analyses. If applicable, describe which groupings were chosen and why	7-8
Statistical methods	12	(*a*) Describe all statistical methods, including those used to control for confounding	7-8
		(*b*) Describe any methods used to examine subgroups and interactions	N/A
		(*c*) Explain how missing data were addressed	9
		(*d*) If applicable, explain how loss to follow-up was addressed	
		(*e*) Describe any sensitivity analyses	
**Results**			
Participants	13*	(a) Report numbers of individuals at each stage of study—eg numbers potentially eligible, examined for eligibility, confirmed eligible, included in the study, completing follow-up, and analysed	9
		(b) Give reasons for non-participation at each stage	9
		(c) Consider use of a flow diagram	N/A
Descriptive data	14*	(a) Give characteristics of study participants (eg demographic, clinical, social) and information on exposures and potential confounders	9-10
		(b) Indicate number of participants with missing data for each variable of interest	N/A
		(c) Summarise follow-up time (eg, average and total amount)	N/A
Outcome data	15*	Report numbers of outcome events or summary measures over time	9-10

For peer review only

Main results	16	(*a*) Give unadjusted estimates and, if applicable, confounder-adjusted estimates and their precision (eg, 95% confidence interval). Make clear which confounders were adjusted for and why they were included	11-15
		(*b*) Report category boundaries when continuous variables were categorized	11-15
		(*c*) If relevant, consider translating estimates of relative risk into absolute risk for a meaningful time period	N/A
Other analyses	17	Report other analyses done—eg analyses of subgroups and interactions, and sensitivity analyses	11
**Discussion**			
Key results	18	Summarise key results with reference to study objectives	16
Limitations	19	Discuss limitations of the study, taking into account sources of potential bias or imprecision. Discuss both direction and magnitude of any potential bias	16-17
Interpretation	20	Give a cautious overall interpretation of results considering objectives, limitations, multiplicity of analyses, results from similar studies, and other relevant evidence	19
Generalisability	21	Discuss the generalisability (external validity) of the study results	16,18
**Other information**			
Funding	22	Give the source of funding and the role of the funders for the present study and, if applicable, for the original study on which the present article is based	20

*Give information separately for exposed and unexposed groups.

Note: An Explanation and Elaboration article discusses each checklist item and gives methodological background and published examples of transparent reporting. The STROBE checklist is best used in conjunction with this article (freely available on the Web sites of PLoS Medicine at http://www.plosmedicine.org/, Annals of Internal Medicine at http://www.annals.org/, and Epidemiology at http://www.epidem.com/). Information on the STROBE Initiative is available at http://www.strobe-statement.org.

For peer review only
